# The Role of White Matter in the Neural Control of Swallowing: A Systematic Review

**DOI:** 10.3389/fnhum.2021.628424

**Published:** 2021-06-28

**Authors:** Ann Alvar, Rachel Hahn Arkenberg, Bethany McGowan, Hu Cheng, Georgia A. Malandraki

**Affiliations:** ^1^I-EaT Swallowing Research Laboratory, Speech Language and Hearing Sciences, Purdue University, West Lafayette, IN, United States; ^2^Libraries and School of Information Studies, Purdue University, West Lafayette, IN, United States; ^3^Psychological and Brain Sciences, Imaging Research Facility, Indiana University, Bloomington, IN, United States; ^4^Weldon School of Biomedical Engineering, Purdue University, West Lafayette, IN, United States

**Keywords:** white matter, swallowing, dysphagia, deglutition, neurophysiology, diffusion weighted imaging, diffusion tensor MRI

## Abstract

**Background:** Swallowing disorders (dysphagia) can negatively impact quality of life and health. For clinicians and researchers seeking to improve outcomes for patients with dysphagia, understanding the neural control of swallowing is critical. The role of gray matter in swallowing control has been extensively documented, but knowledge is limited regarding the contributions of white matter. Our aim was to identify, evaluate, and summarize the populations, methods, and results of published articles describing the role of white matter in neural control of swallowing.

**Methods:** We completed a systematic review with a multi-engine search following PRISMA-P 2015 standards. Two authors screened articles and completed blind full-text review and quality assessments using an adapted U.S. National Institute of Health's Quality Assessment. The senior author resolved any disagreements. Qualitative synthesis of evidence was completed.

**Results:** The search yielded 105 non-duplicate articles, twenty-two of which met inclusion criteria. Twenty were rated as Good (5/22; 23%) or Fair (15/22; 68%) quality. Stroke was the most represented diagnosis (*n* = 20; 91%). All studies were observational, and half were retrospective cohort design. The majority of studies (13/22; 59%) quantified white matter damage with lesion-based methods, whereas 7/22 (32%) described intrinsic characteristics of white matter using methods like fractional anisotropy. Fifteen studies (68%) used instrumental methods for swallowing evaluations. White matter areas commonly implicated in swallowing control included the pyramidal tract, internal capsule, corona radiata, superior longitudinal fasciculus, external capsule, and corpus callosum. Additional noteworthy themes included: severity of white matter damage is related to dysphagia severity; bilateral white matter lesions appear particularly disruptive to swallowing; and white matter adaptation can facilitate dysphagia recovery. Gaps in the literature included limited sample size and populations, lack of in-depth evaluations, and issues with research design.

**Conclusion:** Although traditionally understudied, there is sufficient evidence to conclude that white matter is critical in the neural control of swallowing. The reviewed studies indicated that white matter damage can be directly tied to swallowing deficits, and several white matter structures were implicated across studies. Further well-designed interdisciplinary research is needed to understand white matter's role in neural control of normal swallowing and in dysphagia recovery and rehabilitation.

## Introduction

Swallowing is an essential biological function governed by both peripheral and central sensorimotor pathways. Damage in these pathways can cause swallowing disorders, also known as dysphagia. Dysphagia is a frequent consequence of many neurological and anatomical conditions or diseases (e.g., stroke, cerebral palsy, Parkinson's disease, dementia, head and neck cancer, trauma, etc.), and is very common. In the US alone, four percent of adults are reported to experience dysphagia per year (Bhattacharyya, 2014). For those individuals, the impact can be profound. Dysphagia affects quality of life (Leow et al., 2010), nutrition (Namasivayam and Steele, 2015), hydration (Reber et al., 2019), respiratory function, and overall health (Langmore et al., 1998). Because of its impact and relatively high prevalence, developing effective interventions for the management of dysphagia has been a longstanding goal of clinicians and researchers. Central in these efforts has been the attempt to increase our knowledge and understanding of the underlying physiological and neurophysiological mechanisms that govern swallowing, and which can be targeted in treatment.

This knowledge base has been growing over the past 100 years, with much of the literature focused on the role of cortical and brainstem gray matter areas involved in the neural control of swallowing. In the early 1900's, neuroscience research relied heavily on animal models and focused on the reflexive nature of swallowing (Miller and Sherrington, 1915). This animal work revealed the essential role that brainstem nuclei, specifically a group of medullary nuclei, play in triggering the pharyngeal response (Doty, 1951, 1968; Car and Roman, 1969; Jean et al., 1975; Amri et al., 1984; Kessler and Jean, 1985). In the mid 1900's, some attention was directed to the cortex (Car, 1970; Sumi, 1972), as researchers found that swallowing or mastication were evoked when specific cortical regions (i.e., the lateral pericentral and superior sylvian cortex) were stimulated with electrical pulses in patients under seizure evaluation (Penfield and Welch, 1949; Penfield, 1955). This same response was also seen in animals during intracranial microelectrode stimulation (Sumi, 1972; Martin et al., 1999). Despite these findings, the theory that swallowing is primarily *reflexive* (i.e., brainstem mediated) predominated from the early 1900's even into the 1980's (Bosma, 1957; de Lama Lazzara et al., 1986). During this time period, this notion started being challenged with the advent of new imaging techniques that allowed researchers to non-invasively look at changes in brain structures in living humans.

Clinical studies reporting swallowing deficits in patients with cortical and subcortical lesions provided the first clear support that the role of the cerebrum was essential in swallowing (Meadows, 1973; Gordon et al., 1987; Martin and Sessle, 1993; Robbins et al., 1993; Daniels et al., 1996). This was then further delineated through novel neuroimaging techniques that enabled the study of metabolic correlates of brain activation during swallowing *in vivo* (e.g., Hamdy et al., 1999; Martin et al., 2001, 2004; Suzuki et al., 2003; Toogood et al., 2005; Malandraki et al., 2009, 2011). This growing body of literature was critical in shifting our appreciation of swallowing from a simple brainstem mediated reflex to a highly complex sensorimotor function relying on all levels of the central nervous system (CNS) (Malandraki et al., 2011).

Undoubtedly, identifying the gray matter regions that play a role in swallowing was a significant contribution. However, the specifics on how these regions communicate and connect with each other to achieve this complex control remains largely unexplored, i.e., there is little insight on the role of white matter tracts. One early computed tomography (CT) study reported that damage to subcortical white matter (the internal capsule and within the brainstem) caused dysphagia, likely due to disruption in the sensorimotor pathways of the corticobulbar tract (Logemann et al., 1993). Additional early CT/MRI work showed that lingual discoordination and dysphagia were common in patients with periventricular white matter lesions (Daniels et al., 1999).

Despite the relatively limited focus on the role of white matter for swallowing, it is evident from broader neurophysiology work that white matter is highly relevant to the study of all human functions. White matter is the CNS component composed primarily of myelinated axons of neurons, provides the connections between cells, and functions as the information highway between distinct brain regions. These connections bundle together to form three primary types of white matter tracts. First, association tracts connect areas of the cortex within the same hemisphere (Schmahmann et al., 2007). One prominent association tract is the superior longitudinal fasciculus which contains many branches, most notably the arcuate fasciculus, which connects the Broca's and Wernicke's areas in the left hemisphere (Breier et al., 2008). Damage to these structures in each hemisphere can lead to different symptoms, due to the lateralization of functions. Secondly, commissural tracts are the tracts that connect the right and left hemispheres, and include the corpus callosum, and the anterior and posterior commissures. Damage to these inter-hemispheric structures can cause frontal lobe dysfunction and spatial deficits (Buklina, 2005). Lastly, projection tracts connect areas in the cortex with lower centers such as deep nuclei or the brainstem. An example of projection tracts/structures are the internal capsules, which carry motor and sensory information between the cortex and sub-cortical areas. Damage to projection fibers can result in motor or sensory deficits throughout the body (Puig et al., 2011; Emos and Agarwal, 2020).

Scientific and clinical interest in these white matter tracts has been increasing. In a PubMed search using the key word “white matter,” we identified a 400% increase in relevant articles since 1999. This increase is most likely due to the emergence of a new field devoted to understanding the full network of these tracts in humans, known as the human connectome (Sporns et al., 2005). This field has evolved through imaging advancements in diffusion weighted imaging (DWI), and in analysis techniques, such as tractography (Huisman, 2010). Diffusion weighted imaging senses the diffusion of water across tissue materials. Notably, white matter tracts have a unique diffusion property; they are anisotropic, i.e., water diffuses predominantly along the fiber (Frank, 2001). This characteristic (captured with DWI) allows us to identify and describe white matter structures and their properties with simple metrics such as fractional anisotropy (Alexander et al., 2007).

Although our understanding of the role of white matter for many biological functions is increasing, our understanding of these pathways in the neural control of swallowing remains scarce. Further, it is unclear to what extent newer imaging techniques such as DWI/DTI have been used for the study of the swallowing control. Identifying and addressing these gaps will provide critical insight on the structural neural connections involved in swallowing and has the potential to improve our ability to accurately identify and treat patients with neurological disease and dysphagia. Further, white matter is highly adaptable as shown by studies on recovery of sensorimotor functions after neurotrauma (Schlaug et al., 2009; Kou and Iraji, 2014; Sampaio-Baptista and Johansen-Berg, 2017), and may hold potential for maximizing swallowing recovery, but it is unclear to what extent this has been investigated. As a first step to informing future research in this line of work, it is necessary to systematically evaluate the quality of existing evidence and compare results across studies. Therefore, this systematic review aimed to identify all published research articles describing the role of white matter in the neural control of swallowing, and summarize and evaluate them to determine answers to four primary research questions:

1. What patient populations are represented in the available evidence?
2. What white matter imaging techniques and swallowing evaluation techniques have been utilized to investigate the role of white matter in the neural control of swallowing?
3. Does the available evidence provide definitive information on specific white matter tracts that are implicated in the neural control of swallowing and their role?
4. What are the main gaps in the investigation of the role of white matter tracts in the neural control of swallowing that need to be addressed in future research?

## Methods

### Systematic Review Protocol

This review was conducted systematically and the detailed protocol was developed a priori in accordance with the PRISMA-P 2015 (Preferred Reporting Items for Systematic Reviews and Meta-Analyses) guidelines (Moher et al., 2009). Further, it was registered with the international prospective register of systematic reviews (PROSPERO ID: CRD 42020191453). Throughout the process, Rayyan data management software was utilized for blinding and tracking (Ouzzani et al., 2016).

### Literature Search Strategy

A health sciences librarian (third author, BM) performed literature searches from May 2020 through July 2020, in the following databases: MEDLINE (*via* PubMed), Cochrane Library Database of Systematic Reviews, CINAHL, and Web of Science. Searches included a combination of controlled vocabulary terms, when applicable, and free text keywords. No filters were used during the search process. The literature was searched using combinations of terms including the following: “magnetic resonance imaging,” OR “MRI,” OR “white matter,” AND “deglutition disorders,” OR “dysphagia.” Terms were nominated by the senior/last author (GAM) and were further discussed and agreed upon with the entire team, including an MRI physicist and experienced imager (fourth author; H.C.). Both initial and final searches included a back-chained search of the reference lists of all identified articles. The final search was executed in November 2020, just before manuscript submission, to capture new publications. The precise search strategies, databases searched, and the number of results retrieved per database, are available in Supplementary Table A.

### Inclusion and Exclusion Criteria

Studies were included in this review if they met the following criteria: (1) included human subjects of all ages, (2) were peer-reviewed research articles, scientific abstracts, case studies, or reviews, (3) were written in English language, (4) reported aberration of white matter microstructure/pathways and/or documentation of damage to white matter resulting in dysphagia, (5) used one or more of the following methods: MRI, diffusion MRI (dMRI), diffusion-weighted imaging (DWI), diffusion tensor imaging (DTI), tractography, and/or structural brain network (connectome), and (6) included swallowing/dysphagia as a primary outcome.

Consequently, studies were excluded if they: (1) included only animal models, (2) were not in English, (3) did not specifically document white matter aberration, or (4) did not include swallowing as a primary outcome measure.

The two first authors (AA and RHA) independently screened all titles and abstracts identified with the search strategy for inclusion/exclusion, and any disagreements were resolved by the senior/last author (GAM). Then, the same two authors (AA and RHA) reviewed the full text of all qualifying articles to make the final decision of eligibility, with disagreements, again, resolved by the senior/last author (GAM).

### Bias Assessment

In order to compare results across studies, it is essential to assess study quality and risk of bias. Because the majority of the studies identified in this review were observational cohort studies, study quality was assessed using an adapted version of the U.S. Department of Health and Human Services National Institute of Health's (NIH) Quality Assessment protocol for observational cohort and cross-sectional studies (National Heart, Lung, and Blood Institute, 2019). This 12–item tool was developed to assist reviewers in critically appraising the internal validity of studies through a structured evaluation of sources of bias, diagnosis and outcome measures, and statistical components. For the purpose of this study, three of 12 original assessment items in the protocol were not considered in the quality assessment, because they were consistently not reported or not applicable in the studies reviewed (these were: sufficient timeframe to see exposure effect, repeated exposure assessment, and follow-up rate). See Table 1 for a full list of items assessed, and Supplementary Table B for details on the final adapted quality assessment tool.

**Table 1:** Study quality parameters rated using the modified NIH quality assessment for observational cohort and cross-sectional studies.

**First author date**	**Research question and hypotheses**	**Study population**	**Uniform eligibility criteria**	**Sample size justification**	**Primary diagnoses determined before outcome**	**Different levels of severity of diagnosis**	**Diagnosis measures (white matter integrity)**	**Outcome measures (swallowing)**	**Blinding of outcome measures**	**Statistical analysis (confounding variables)**	**Overall rating**
Cola et al. (2010)	+	+	+	−	+	+	+	+	+	+	Good
Galovic et al. (2013)	+	+	+	−	+	+	+	p	CD	+	Good
Galovic et al. (2016)	+	+	+	−	+	+	+	+	−	p	Good
Mihai et al. (2016)	+	+	+	+	+	+	+	p	−	p	Good
Wilmskoetter et al. (2019)	+	+	+	+	+	+	+	+	CD	+	Good
Jang et al. (2020b)	+	+	+	−	+	−	+	+	−	−	Fair
Jang et al. (2020a)	+	+	+	−	+	−	+	+	−	−	Fair
Lee et al. (2020)	+	+	+	−	+	+	p	p	CD	+	Fair
Fandler et al. (2018)	+	+	+	−	+	+	+	p	−	−	Fair
Fandler et al. (2017)	p	+	+	−	+	+	+	p	CD	+	Fair
Flowers et al. (2017)	+	+	+	−	CD	+	p	−	CD	+	Fair
Galovic et al. (2017)	p	+	+	−	+	−	+	+	+	p	Fair
Jang et al. (2017)	+	+	NA	NA	+	+	+	−	−	−	Fair
Ko et al. (2019)	p	+	+	−	+	+	p	p	CD	+	Fair
Kumar et al. (2012)	+	+	+	−	CD	+	p	p	CD	+	Fair
Levine et al. (1992)	p	+	+	−	NA	+	p	p	CD	p	Fair
Li et al. (2014)	+	+	+	−	+	−	+	+	CD	−	Fair
Moon et al. (2017)	p	+	+	−	+	+	+	p	−	+	Fair
Mouräo et al. (2017)	+	+	+	−	+	+	+	p	−	p	Fair
Suntrup et al. (2015)	p	+	+	−	+	+	p	p	+	−	Fair
Kim et al. (2014)	p	−	+	CD	CD	−	p	+	−	−	Poor
Wan et al. (2016)	p	+	+	−	CD	−	−	+	CD	−	Poor

*+ yes (adequately addressed), −no (not adequately addressed), p, partially addressed; CD, cannot determine; NA, not applicable*.

Each study was independently evaluated by the two first authors (AA and RHA) using the NIH Quality Assessment protocol and was given a cumulative rating of Good, Fair, or Poor, that summarized the risk of bias in the study. According to the NIH Quality Assessment guidelines for the cumulative ratings, research studies are rated as “Good” if they have the least risk of bias, although they may not be free from all potential biases (Study Quality Assessment Tools | NHLBI, NIH, 2019). Bias in papers rated as “Good” is minimal and is discussed and accounted for in analysis. A study rated as “Fair” is still considered valid, with useful information, but may have some clear risk of bias that is not addressed. For example, a fair study may be underpowered due to limited sample size and not including blinding, both of which increase risk of bias. Finally, studies rated as “Poor” have substantial methodological limitations across multiple categories that limit interpretation of results. This tool does not determine precise cut-offs between these quality categories, but instead helps the evaluators rate the overall risk of internal bias based on key items/questions (see Supplementary Table B). To maximize objectivity, we used two independent raters (first two authors, AA and RHA), who participated in a 3-h practice training on this tool led by the senior author (GAM), before starting its use. Any disagreements in quality ratings were planned to be resolved through discussion with the senior/last author (GAM), though no disagreements occurred.

### Data Extraction and Qualitative Synthesis

After completion of the bias assessment, the two first authors (AA and RHA) independently extracted data from all articles. Information extracted from each paper included study type and population characteristics, white matter techniques and information, swallowing evaluation methods, outcome measurements, and main findings from each study.

Extracted data on study type and population characteristics included: study design [classified in accordance with (Mann, 2003)], number of patients, number of participants in control group (if applicable), underlying diagnosis/disease of patient group or subgroups, severity/state of disease, age, sex, race, and ethnicity. Data extracted on white matter measurement included: scan type, scanner model and strength, head coil type, b-values, and number of directions (for DWI scans), scan settings, analysis type, and analysis method.

We also extracted details on how the primary outcome variable (swallowing) was assessed in order to compare clinical findings across studies. Data extracted included swallowing measurement method (e.g., instrumental or clinical assessment) and analysis of swallowing components (e.g., use of Penetration Aspiration Scale (Rosenbek et al., 1996), temporal measures, binary clinical ratings, etc.). Finally, information was extracted on the studies' main findings, which included implicated white matter areas and their suggested role in swallowing (including the statistical descriptions of that relationship), and limitations of each study.

Due to the wide variety of study types, patient populations, and imaging and swallowing evaluation methods, it was not possible to analyze the data across studies quantitatively at this time. Instead, a qualitative synthesis of the findings across studies was completed while critically evaluating the risk of bias of their methods and results.

## Results

### Study Selection and Data Extraction

**Figure 1:**
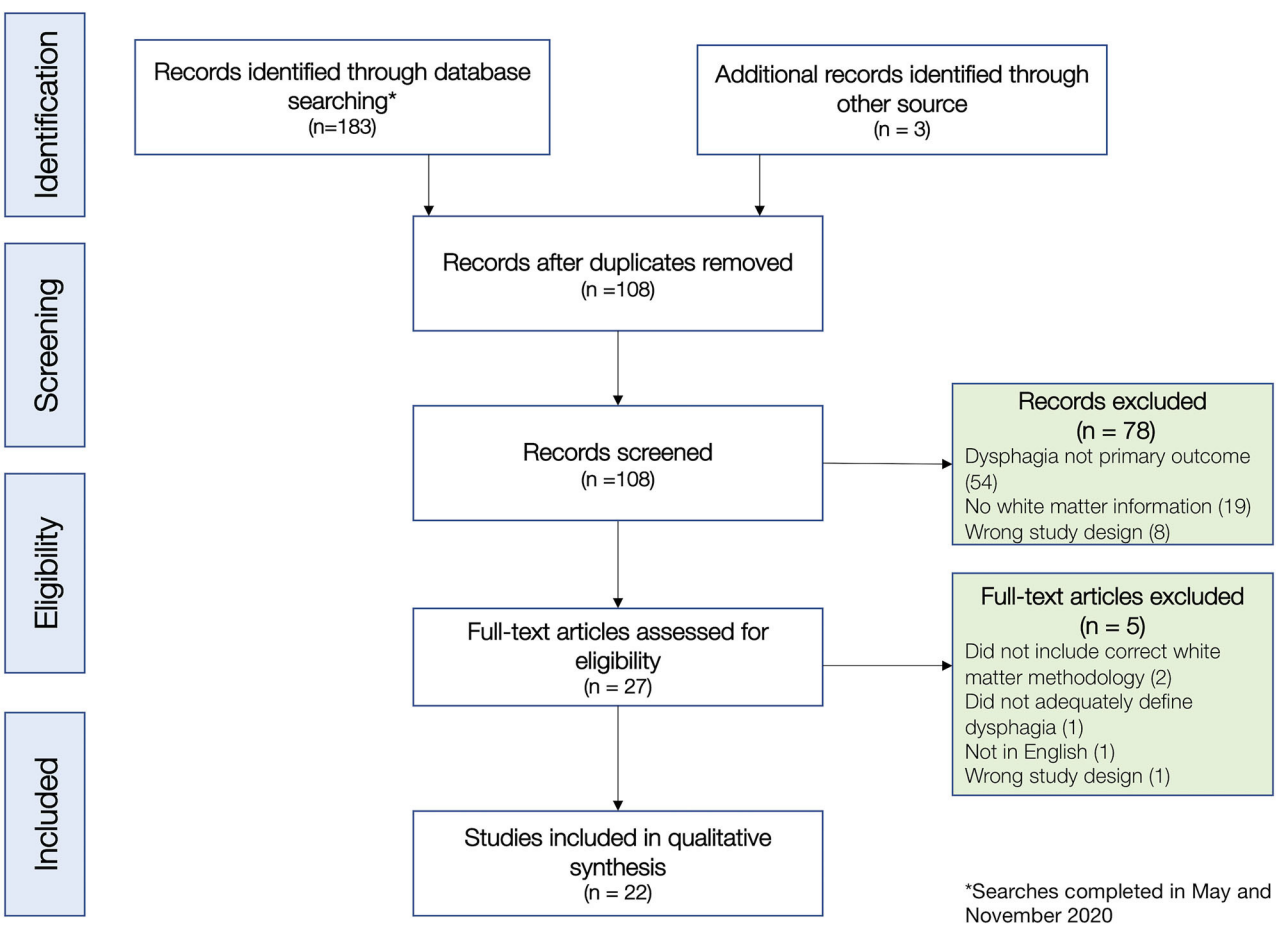
Study selection flow diagram, according to PRISMA standards.

The initial search of four databases in May-July 2020 retrieved a total of 182 titles, including 78 duplicates and 104 unique titles. Two articles were identified *via* backward citation chaining, and two additional articles (Jang et al., 2020b; Lee et al., 2020) were identified in a search verification in September 2020. A final search was conducted on November 8th, before submission, which revealed three novel articles, none of which met inclusion criteria. In total, this resulted in a subset of 108 non-duplicate articles, which all had full abstracts. Two independent reviewers (first two authors; AA and RHA) reviewed the titles and abstracts, and after this review, 27 articles met our inclusion criteria for full text review. Following a detailed full text review, 22 articles were selected for inclusion (see Figure 1 for CONSORT diagram and exclusion reasons). There was disagreement on three articles at the screening stage, which was resolved by the senior/last author (GAM), and there were no other author disagreements on article review and inclusion. After study selection, the two first authors (AA and RHA) extracted data independently with agreement on 91% of extracted items and reached consensus on the remaining 9% of items extracted.

### Study Types and Characteristics

Of the 22 articles included in the review, one was a case study (Jang et al., 2017), and 21 included multiple participants and were observational, i.e., there was no intervention assessed (Table 2). The most frequent observational study design employed was retrospective cohort (*n* = 11), followed by three case control studies, and one prospective cohort study. Six studies did not neatly fit into one of the main observational study categories (Mann, 2003). Four of these were closest to a prospective cohort design (Li et al., 2014; Suntrup et al., 2015; Galovic et al., 2016, 2017). However, instead of examining whether an outcome of interest (i.e., dysphagia) would develop *over time*, these four studies included only participants who had *already* failed a dysphagia screening or were diagnosed with dysphagia. One study identified a convenience sample with a common diagnosis (cerebral palsy, CP) and used observational methods to determine dysphagia status (Mouräo et al., 2017), and one study recruited healthy individuals to look at both a risk factor (i.e., aberrations in white matter) and changes in swallowing (Levine et al., 1992). Because these six studies did not fit into a specific pre-defined study type, but all involved carefully defined groups of subjects, we identified them broadly as “cohort design.”

**Table 2:** Demographics and participant characteristics across studies.

	**Number of participants (groups)**	**Age and sex**	**Race and ethnicity**	**Inclusion criteria and underlying diagnosis**	**Clinical rating of diagnosis**
Jang et al. (2020b) Retrospective Cohort	40 (20 controls; 16 pts w/dysphagia and NGT <6 mos; 4 pts w/dysphagia and NGT > 6 mos)	Control: 56 ± 10 (55% female) NGT <6 mos: 60.6 ± 12.4 (37.5% female). NGT > 6 mos: 63.2 ± 14.7 (75% female)	NR	Stroke: Lateral medullary infarct with dysphagia and NGT placement	NR
Jang et al. (2020a) Retrospective cohort	64 (22 controls; 42 pts w/dysphagia; 10 pts w/dysphagia and NGT <2 days; 27 pts w/dysphagia and NGT <6 mos; 5 pts w/dysphagia with NGT > 6 mos)	Control: 51 ± 11 (50% female) NGT <2 days: 51 ± 15 (40% female) NGT <6 mos: 60 ± 8.9 (52% female) NGT > 6 mos: 59 ± 10 (40% female)	NR	Stroke: Supratentorial intra-cerebral and intra-ventricular hemorrhage with dysphagia and NGT placement	NR
Lee et al. (2020) Retrospective cohort	137	68.7 ± 14 (49.6% female)	NR	Stroke: acute ischemic stroke and referred for VFSS	NIHSS
Ko et al. (2019) Case control	87 (20 pts with LA in contralateral CBT; 67 LA not in ipsilateral CBT)	64.6 ± 11.5 (36% female)	NR	Stroke: First unilateral corona radiata infarct involving CBT with LA	NIHSS, Korean mini mental, motricity index of limbs
Wilmskoetter et al. (2019) Retrospective cohort	68	68.21 ± 15.23 (53% female)	White 45 (66%), Black 21 (31%), Asian 1 (1.5%), Other 1 (1.5%); Not Hispanic/Latino: 68 (100%)	Stroke: First (and only) hemispheric stroke of the MCA	NIHSS (mean 12.57 ± 6.97), Rankin Scale (median 0, range 0–4)
Fandler et al. (2018) Retrospective cohort	243 (196 pts w/o dysphagia; 47 pts w/dysphagia)	Pts w/o dysphagia: 67.1 ± 12.4 (37.2% female) Pts w/dysphagia: 70.8± 10.8 (30% female);	NR	Stroke: Recent small subcortical infarct	NIHSS 0–4 (263, 79.2%), NIHSS>5 (69, 20.88%)
Fandler et al. (2017) Retrospective cohort	322 (249 pts w/o dysphagia; 83 pts w/dysphagia)	Pts w/o dysphagia: 67 ± 12.4 (7.2% female) Pts w/dysphagia: 70.8 ±10.8 (29.8% female)	NR	Stroke: Recent small subcortical infarct	NIHSS: Dysphagia 3 (1–10) No dysphagia 3 (0–9)
Flowers et al. (2017) Retrospective cohort	160 (84 pts w/o dysphagia; pts w/76 dysphagia)	Total: 66.7 ± 15 (43.1% female) Pts w/o dysphagia: 63.6 ± 15.6 (46.4% female) Pts w/dysphagia: 69.9 ± 13.8 (39.5% female)	NR	Stroke (no other specifications)	Rankin scale, CNS score
Galovic et al. (2017) Cohort	62 pts (24 additional controls for white matter modeling)	Pts: 75 ± 21 years (55% female). Controls: 63 ± 9 (42% female)	NR	Stroke: First hemispheric stroke leading to impaired oral intake	NR
Jang et al. (2017) Single case study	1 pt (3 additional controls for modeling)	59 (Male)	White	Stroke (no other specifications)	NR
Moon et al. (2017) Retrospective cohort	63 (49 mild lesions, 14 severe lesions)	77.24 ± 7.22 (50.8% female)	NR	Stroke: Mild first stroke, had dysphagia symptoms and completed VFSS.	NIHSS-K <5, mean = 2.83, SD = 1.42
Mouräo et al. (2017) Cohort	20 (13 left hemisphere; 7 right hemisphere)	5.11–17.6 (45% female)	NR	Unilateral spastic cerebral palsy	GMFCS, MACS
Galovic et al. (2016) Cohort	119 (107 tube independent; 12 tube dependent)	Tube independent: 71 ± 19 (45% female) Tube dependent: 76 ± 9 (50% female)	NR	Stroke: First hemispheric stroke who failed dysphagia screening	NIHSS
Mihai et al. (2016) Case control	36 (18 patients; 18 control)	Pts: 56.6 ± 15.3 (27.8% female) Control: 61.94 ± 9.78 (77.8% female)	NR	Stroke: Single ischemic stroke (recovered from Severe dysphagia w/in last 3 years)	NR
Suntrup et al. (2015) Cohort	200 (35 pts w/o dysphagia; 85 pts w/mild dysphagia; 80 pts w/severe dysphagia)	73.7 years (49.5% female) Pts w/o dysphagia: (72 ± 10.7) Pts w/mild dysphagia: (73.5 ± 13.1) Pts w/severe dysphagia: (74.8 ± 11.9)	NR	Stroke: First stroke; failed dysphagia screening and completed FEES	NIHSS
Li et al. (2014) Cohort	36 (12 pts w/dysphagia; 12 pts w/o dysphagia; 12 controls)	Pts w/o dysphagia: 66.5 ± 5.2 (41.7% female) Pts w/dysphagia: 65.2 ± 4.3 (50% female) Control: 65.8 ± 3.3 (50% female)	NR	Stroke: First (and only) hemispheric stroke of the MCA	NR
Galovic et al. (2013) Prospective Cohort	94 (34 acute risk; 60 no risk; 7 days later within the acute risk group: 17 transient risk; 17 extended risk)	Acute risk: 74 ± 19 (59% female) No risk: 71.5 ± 16 (43% female)	NR	Stroke: First	NIHSS, Rankin scale
Kumar et al. (2012) Retrospective cohort	77	Median = 76 (64.9% female)	NR	Stroke: Acute ischemic stroke and severe dysphagia	NIHSS (median 8)
Cola et al. (2010) Case Control	45 (10 RHD; 10 LHD; 25 control)	RHD: 62.3 ± 12.1 (10% female) LHD: 62.3 ± 8.7 (0% female) Control: 67.2 ± 9.1(8% female)	RHD: 7 black (70%) LHD: 8 black (80%) Control: 4 black (16%)	Stroke: Acute unilateral ischemic stroke	NIHSS
Levine et al. (1992) Cohort	49 controls (25 MRI score 0-1; 24 MRI score 2-3)	Mean = 66 (40.8% female)'	NR	NA	NA
^*^Wan et al. (2016) Retrospective cohort	12	66 ± 10 (25% female)	NR	Stroke: Basal ganglia and/or centrum semiovale	NR
^*^Kim et al. (2014) Retrospective cohort	103 (62 anterior infarcts; 19 posterior infarcts; 22 WM disease)	NR	NR	Stroke: First unilateral ischemic stroke within 3 months; had dysphagia symptoms and completed VFSS in last 3 months	NR

* *Received a modified NIH Quality Assessment rating of “poor”.*

*Order of appearance: pts, patients; w/with; mos, months; w/o, without; NGT, naso-gastric tube; NR, not reported; NIHSS, national institute of health stroke scale; NIHSS-K, national institute of health stroke scale Korean version; VFSS, videofluoroscopic swallow study; RHD, right hemisphere disease; LHD, left hemisphere disease; MRI, magnetic resonance imaging; LA, leukoaraiosis (hyperintensity around ventricles); CBT, corticobulbar tract; CNS, Canadian Neurological Scale; GMFCS, gross motor function classification scale; MACS, manual ability classification scale; NA, not applicable*.

### Participant Characteristics and Clinical Classifications

Demographic and clinical diagnosis data (Research Question 1) of all reviewed studies are also included in Table 2. Ages of participants ranged from five to 96 years old, and only one study included patients under 18, children with CP, ages 5.11-17.6 (Mouräo et al., 2017). One study did not report age of the participants (Kim et al., 2014). Only two studies reported race (Cola et al., 2010; Wilmskoetter et al., 2019), and one also reported ethnicity (Wilmskoetter et al., 2019). The number of participants across studies ranged from a single subject to 322 (mean = 92.77), and 40.88% of all subjects were female, though one study did not report sex (Kim et al., 2014). One article included exclusively healthy participants, and 21 articles included patients. Of these 21 articles, seven also included groups of healthy adults, four as control groups and three for white matter mapping and modeling, not for comparison.

The majority of subjects were adult patients post stroke (20 articles) and, as already mentioned, one study focused on children with CP (Mouräo et al., 2017). Thirteen studies included a clinical rating of the underlying diagnosis of their subjects (Table 2). For stroke, the most frequent clinical rating scale used was the National Institute for Health Stroke Scale (NIHSS; *n* = 10). Three studies used the Rankin Scale (*n* = 3), one used the Canadian Neurological Scale (*n* = 1), one used the Korean mini mental (*n* = 1), and one used the motility index of limbs (*n* = 1) (Table 2). The clinical ratings used for CP were the Gross Motor Function Classification System (GMFCS) and the Manual Ability Classification System (MACS) (Mouräo et al., 2017).

Notably, within the studies that focused on stroke (20/22), inclusion criteria varied substantially. One study listed broad criteria of stroke diagnosis without further specification, eleven required that it was the first stroke, six required damage to a specific region, such as the middle cerebral artery, and nine required varying degrees of dysphagia severity for inclusion (Table 2).

### Imaging Parameters and Analysis

#### Data Acquisition

Imaging specifications (Research Question 2) used in all reviewed studies are detailed in Table 3. All studies used MR imaging, however, specifics on scanner models and strength, scan types, settings, and analysis methods varied.

**Table 3:** MR imaging specifications across studies.

	**Scan type**	**Scanner model and head coil**	**Diffusion settings and directions**	**MRI scans settings**	**Analysis type**	**Analysis specifications**
Jang et al. (2020b)	DWI	1.5T Phillips Gyroscan Intera6 channel head coil	b = 1,000 s/mm^2^	Acquisition matrix = 96 × 96; reconstructed matrix = 192 × 192; FOV = 240 × 240 mm; TR = 10,398 ms; TE = 72 ms; EPI factor = 59; slice thickness=2.5 mm	Seed based Tractography FA; TV; Fazekas Grade	FMRIB Diffusion Software with routines option (0.5 mm step lengths, 5,000 streamline samples, curvature threshold = 0.2) used for fiber tracking
Jang et al. (2020a)	DWI	1.5T Phillips Gyroscan Intera6 channel head coil	b = 1,000 s/mm^2^	Acquisition matrix=96 × 96; reconstructed to matrix = 192 × 192; FOV = 240 × 240 mm^2^; TR = 10,398 ms; TE = 72 ms; SENSE factor = 2; EPI factor = 59; slice thickness = 2.5 mm.	Seed based Tractography FA; TV; Modified Graeb Score	FMRIB Diffusion Software with routines option(0.5 mm step lengths, 5,000 streamline samples, curvaturethresholds = 0.2) used for fiber tracking; analysis done by expert w/3 years of experience
Lee et al. (2020)	DWI, FLAIR	NR	NR	NR	Fazekas grade (manual rating)	Two physiatrists completed analysis
Fandler et al. (2017)	T2- axial fast spin echo, axial T2 FLAIR, sagittal T1 spin echo, gradient echo T2, axial diffusion-weighted single shot echo planar axial	1.5T Siemens Symphony	NR	All axial slices slice thickness = 5 mm	Manual lesion identification; Fazekas Grade	Two independent experts used standards for reporting vascular changes on neuroimaging consensus criteria
Fandler et al. (2018)	T2- axial fast spin echo sequence, FLAIR sequence–axial, gradient echo T2, DWI single-shot echo planar with ADC maps (axial) and TOP angiography	1.5 T	NR	Axial T2-weighted fast spin echo sequence (0.5 × 0.5 × 5 mm); FLAIR sequence (0.4 × 0.4 × 5 mm); sagittal T1-weighted spin echo sequence (0.6 × 0.6 × 5 mm); gradient echo T2^*^ weighted sequence (0.4 × 0.4 × 5 mm); axial DWI single-shot echo planar imaging sequence (1.2 × 1.2 × 5 mm) with apparent diffusion coefficient ADC maps and a 3D time of flight (TOF) angiography. Axial slices; slice thickness = 5 mm with 0.5 mm gap	Lesion probability mapping; tractography analysis	RSSI manually marked by two neuroimaging experts
Moon et al. (2017)	T2 FLAIR and DWI	NR	NR	NR	Fazekas scale (using diffusion images with FLAIR MRI); unspecified localization	Physician rating
Li et al. (2014)	Resting state fMRI, DTI	3T MRI GE Signa EXCITE	b = 1,000 s/mm^2^ reference scan with b = 0 (no diffusion gradient) 15 non-collinear directions	NR	Seed-based functional connectivity maps (from primary motor and supplementary motor to the brain); swallowing-related functional connectivity for 20 ROIs; mean FA between the SMA and M1	Whole brain fiber tracking using “Diffusion Toolkit” software
Mihai et al. (2016)	DWI, T1-weighted, fMRI	3T Siemens Verio, 32-channel head coil	b = 1,000 s/mm^2^; 64 gradient directions	Gradient echo (34 slices 2 × 2 × 2 mm); functional EPI (96 × 96 oblique); structural T1 (1 × 1 × 1)	FA lateralization index; T1 lesion size	FSL, MNI, and bedpostx were used for probabilistic tractography; researchers manually drew borders for lesions for lesion size, for detailed description of other programs used see paper.
Galovic et al. (2017)	Transverse T2, T1, FLAIR, saggital T2, isotropic DWI	I.5T Siemens Avanto, 1.5T Siemens Symphony, or 3T Siemens Verio MRI	DWI (b = 1,000 s/mm^2^ with 4 mm transverse slices)	FLAIR slice thickness = 5 mm; T2 sagittal slice thickness = 4.5 mm; transverse DWI slice thickness = 4 mm	Voxel-based lesion symptom mapping (VLSM); ROI analysis; Probabilistic Tractography for healthy individuals	VLSM: ICBM standard brain template, NPM software.Tractography: FMRIB Diffusion toolkit, FSL FLIRT algorithm
Flowers et al. (2017)	Saggital T1, T2 FLAIR, isotropic axial diffusion	1.5 T Signa EchoSpeech MR scanner (GE), quadrature head coil	b = 1,000 s/mm^2^	T1: 7.5 mm slice thickness 2 mm space; T2: 5 mm slice thickness = 2 mm spacing; diffusion slice thickness = 5 mm slice w/0 mm spacing	Fazekas scale for periventricular hyperintensities and deep hyperintensities in 12 ROIs	Manually traced lesions on each DWI slice with MRIcron and calculated volumes
Galovic et al. (2013)	T2, T1, FLAIR, Saggital T2, isotropic DWI, with TOF sequence	1.5T Siemens Avanto 1.5T Siemens Symphony or 3T Siemens Verio	b = 1,000 s/m^2^	FLAIR slice thickness = 5 mm; sagittal T2 slice thickness = 4.5 mm; DWI slice thickness = 4 mm	Lesion mapping with ROI	MICRON with MNI space for model. Semi-automatic image analysis by 1 neurologist using MIPAV to Talairach (used Broadmann areas) and visually noted lesions with binomial scale. ImageJ used for lesion size. Age-related white matter documented by Wahlund et al. (2001)
Wilmskoetter et al. (2019)	DWI	NR	NR	Voxel-wise resolution ranged from 0.9375 × 0.9375 × 3 mm to 1.4458 × 1.4458 × 6 mm	Manually drawn lesions; VLSM; ROI when there were no significant findings from VLSM	Lesions drawn by a researcher using MRIcron; reviewed by neurologist with expertise in VLSM; custom MATLAB script for lesion symptom mapping
Levine et al. (1992)	T2	1.5T GE Signa	n/a	NR	T2 scales graded according to Awad et al. (1986); number of unidentified bright objects	NR
Kumar et al. (2012)	DWI	NR	NR	NR	Lesion volume and location on DWI	Image J; brain atlas
Jang et al. (2017)	DTI (at both 5 and 9 weeks)	1.5T Philips Gyroscan Intera	1,000 s/mm^2^	60 continuous slices; 76 ms; 2.5 mm thickness for each of the 32 gradients	Probabilistic tractography; ROI	FSL FMRIB
Cola et al. (2010)	DWI	NR	b = 1,000 s/mm^2^; 3 directions	Slice thickness = 6 mm axial images;	Lesion volumes semi-manually tracked	Image J (semiautomatic threshold)
Ko et al. (2019)	NR	NR	NR	Slice thickness = 3-5 mm	Lesion size using Sims et al. (2009) method; Fazekas scale	Expert
Mouräo et al. (2017)	T1 NPRAGE, MRI, DTI	3T Siemens Magnetom Trio; 32-channel head coil	b = 800 s/mm^2^; 64 directions	T1 and DTI: slice thickness = 2 mm, 75 slices	Tractography; fractional anisotropy; radial diffusivity; mean diffusivity; fibers count	DTI studio
Suntrup et al. (2015)	DWI, T2 FLAIR,	MRI: 1.5T Intera Gyroscan, Philips. CT: somatom Definition AS+ Siemens	b = 1,000 s/m^2^^**^	MRI: slice thickness = 5 mm transverse. reconstruction, 1 mm increment	Atlas-based regional analysis (% of brain affected)	Completed by neuroradiologist using FSL FLIRT, FMRIB and FNIRT
Galovic et al. (2016)	T2 (transverse and sagital), T1, FLAIR, and DWI	1.5T Siemens Avanto, 1.5T Siemens Symphony or 3T Siemens Verio	b = 1,000 s/m^2^	DWI transverse slices 4 mm; T2: T1: FLAIR slice thickness = 5 mm; Sagittal T2: slice thickness = 4.5 mm	Voxel-based lesion symptom mapping (VLSM)	VLSM was calculated using MRIcron software
Wan et al. (2016)^*^	MRI (no details given)	NR	NR	NR	NR, Though presence of stroke was determined by scans	NR
Kim et al. (2014)^*^	MRI (no details given)	NR	NR	NR	Determined vascular territory with modified Rovira et al. (2005) method	NR

* *Received a modified NIH Quality Assessment rating of “poor”.*

** *The authors reported this as “b = 0 and 1.9 mm/s^2^” which we standardized to b = 1,000 s/m^2^.*

*Order of appearance: DWI, diffusion weighted imaging; FA, fractional anisotropy; TV, tract volume; FMRIB, Functional Magnetic Resonance Imaging of the Brain; FLAIR, fluid attenuated inversion recovery; NR, not rated; RSSI, MRI magnetic resonance imaging; fMRI, functional magnetic resonance imaging; DTI, diffusion tensor imaging; SM, Supplementary motor area and M1; EPI, Echo-planar imaging; FSL, FMRIB Software Library; MNI, Montreal Neurological Institute; VLSM, voxel-based lesion symptom mapping; ICBM, International Consortium for Brain Mapping; NPM, FLIRT FMRIB's Linear Image Registration Tool; MR, Magnetic resonance; MPRAGE, magnetization-prepared rapid gradient-echo; ADC, Apparent Diffusion Coefficient; FACT, Fiber Assignment by Continuous Tracking, apparent diffusion coefficient A*.

Scanner strength is reported in tesla (T), and scanners with higher tesla values allow for a stronger MR signal and may increase the speed of scan acquisition. Fourteen of the 22 reviewed studies provided information on scanner strength (Table 3). Of those 14, three reported using exclusively 3T MRI scanners, two of which were Siemens models, and one GE. Eight reported using exclusively 1.5T, including models from GE (2), Siemens (1), and Philips (4). Three studies utilized scans from both 1.5 and 3T Siemens scanners. Finally, eight studies did not report scanner strength, brand, or model. Only five studies reported information on head coils utilized. Two reported using a 6 channel coil, two reported using a 32 channel coil, and one reported using a quadrature head coil.

In addition to scanner strength and types, a wide variety of scan sequences and parameters were also reported across studies (Table 3), with three studies not reporting scan types at all. Of the studies that did report scan types (19/22), ten acquired a T2-weighted scan (most commonly a T2 FLAIR sequence), and seven a T1-weighted scan. Eighteen studies reported acquiring a DWI/DTI sequence, i.e., the state-of-the-art non-invasive technique to explore white matter integrity (Chanraud et al., 2010). However, it is noteworthy that DWI/DTI scans were not necessarily collected for the studies' white matter analysis, but as standard-of-care protocols for stroke patients (Leiva-Salinas and Wintermark, 2010).

Of the 18 papers that acquired a DWI/DTI sequence, eleven reported their b values (i.e., the diffusion-sensitive gradient factor that helps create different types of contrast between tissues). The most frequently reported b-value was 1,000 s/mm^2^ which has been reported to be the optimal b-value for assessing stroke in the acute phase (Kingsley and Monahan, 2004). Additionally, one pediatric study (CP) reported a b-value of 800 s/mm^2^ (Mouräo et al., 2017).

Another important parameter to consider when acquiring DWI scans is the number of directions in which diffusion is measured (i.e., the greater the number of directions, the greater the number of details that can be mapped in tractography) (Vos et al., 2016). Of the eighteen studies that reported acquiring DWI scans, only four reported the number of directions. Two reported 64 directions, one reported 15, and one three directions (see Table 3).

#### Data Analysis

As expected, given the variety of scan models and specifications utilized, there was also considerable variability in the methods used for white matter data analysis. However, some common themes emerged. The major observation was that researchers followed one of two paths for analysis. Either they examined the **damage/lesions** to white matter by investigating the size and characteristics of these lesions; or they evaluated **white matter integrity** by measuring and describing characteristics of the tissue. Most studies followed the first approach and used qualitative rating scales or quantitative measurements to measure lesions. Studies using this (lesion-based) approach are discussed first followed by studies using the second option (white-matter integrity).

##### Lesion-based analysis: qualitative methods

Eight studies utilized qualitative scales to describe damage to white matter. The most commonly cited scale (*n* = 6) (Fandler et al., 2017; Flowers et al., 2017; Moon et al., 2017; Ko et al., 2019; Jang et al., 2020b; Lee et al., 2020) was the 4-point Fazekas scale (Fazekas et al., 1987). To determine ratings using the Fazekas scale, clinicians or researchers visually examine the amount and size of white matter hyperintensities [i.e., brighter spots on T2-weighted scans that relate to damage to small blood vessels or decrease in myelination (Wardlaw et al., 2015)] in two domains: the periventricular white matter and the deep white matter, and provide a rating of these hyperintensities. An earlier study (Levine et al., 1992) used another 4-point/grade scale developed by Awad et al. using size, multiplicity, and location to rate subcortical incidental lesions (Awad et al., 1986). Further, one study employed a rather indirect approach (Kim et al., 2014), using an adapted scale which categorizes damage to the brain's vascular territories (Rovira et al., 2005), to indirectly infer lesions to white matter.

##### Lesion-based analysis: quantitative methods

Ten of the 22 reviewed studies used a quantitative volume and voxel-based approach to record the location and size of lesions. These studies quantified damage by measuring lesion volume with voxels or mm^2^ lesioned (Galovic et al., 2013, 2016, 2017; Suntrup et al., 2015; Mihai et al., 2016; Flowers et al., 2017; Fandler et al., 2018; Ko et al., 2019; Wilmskoetter et al., 2019; Jang et al., 2020a) or they used these units to derive the amount of area or proportion (%) of an area that is lesioned (Suntrup et al., 2015). Three studies used a technique called voxel-based lesion symptom mapping (VLSM) (Bates et al., 2003), which enables calculation of correlations between locations of the “damaged” voxels and behavioral scores (Galovic et al., 2016, 2017; Wilmskoetter et al., 2019).

##### White matter integrity analysis

Seven of the 22 studies included in this review utilized analysis techniques that provided information on the structural integrity of the white matter, instead of focusing only on lesions. The main quantitative measure reported was fractional anisotropy (FA; *n* = 5) (Table 3). Higher FA values are associated with greater white matter integrity, due to more coherent diffusion of water across tissue (De Erausquin and Alba-Ferrara, 2013). Six studies generally reported using tractography, an analysis method that creates a 3D map of the white matter tracts in the brain, and two of the seven studies measured tract volumes (TV). Of those six, only one reported additional diffusion-based analysis measures, including radial diffusivity, mean diffusivity, and fibers count (Mouräo et al., 2017). These measures give more in-depth information on the integrity of white matter, such as strength of connection and anisotropy of the diffusion (Mori and Zhang, 2006; Clark et al., 2011; de Figueiredo et al., 2011). One study used a different quantitative approach that indirectly informs us about connections in the brain, known as functional connectivity (Li et al., 2014), using the synchrony of the blood oxygen level dependent signals between areas of gray matter to give us indirect insight about how these areas are connected.

### Swallowing Evaluation Methods and Analysis

#### Swallowing Evaluation Methods

All, but one study (Jang et al., 2017), reported details on how swallowing was evaluated (Research Question 2; Table 4). It is commonly accepted that the gold standard for comprehensively evaluating swallowing involves radiologic or endoscopic imaging, i.e., Videofluoroscopic Swallow Studies (VFSS) or Fiberoptic Endoscopic Evaluations of Swallowing (FEES). These evaluation methods allow differing degrees of visualization of the oropharyngeal area, upper airway and upper esophagus, and allow clinicians to make subjective judgments or objective measurements of symptoms, and/or kinematic, temporal and bolus flow events. Fifteen of the 21 studies that reported swallowing evaluation methods used some imaging modality (VFSS and/or FEES; Table 4). Specifically, nine reported use of VFSS for all patient participants, and four reported using VFSS for a subset of subjects (e.g., they included some patients who had received a VFSS while hospitalized post-stroke and others who had only received clinical bedside swallowing evaluations). FEES was used in three studies: one used only FEES (Suntrup et al., 2015), one used FEES in addition to VFSS (Wan et al., 2016), and a third study used FEES for a subset of subjects (Galovic et al., 2017).

**Table 4:** Summary of methods, major findings and limitations of studies included in qualitative synthesis (*n* = 20 studies).

**Study type**	**Underlying diagnosis**	**White matter method**	**Swallow measurement method**	**Swallowing analysis**	**Implicated WM areas**	**Associations with swallowing**	**Limitations**
Jang et al. (2020b) Retrospective cohort	Stroke: lateral medullary w/dysphagia and NGT	DWI FA TV Fazekas grade	VFSS	PAS; FOIS	Corticobulbar tract	FA in CBT was significantly reduced in pts w/longer compared to shorter NGT use or controls (*p* <0.05), but was not significantly different between patients w/dysphagia and NG for <6 mos and controls. Fazekas ratings were not significantly different between patient groups.	CD blinding; did not account for confounding variables in statistical analyses; no sample size justification
Jang et al. (2020a) Retrospective cohort	Stroke: supratentorial intra-cerebral and intra-ventricular hemorrhage w/dysphagia and NGT	DWI FA TV	GUSS, VFSS	PAS; residue scale	Corticobulbar tract	Patients who had NGT removed w/in 2 days had milder CBT injury (only FA decreased, not TV). FA in CBT for each patient group was lower than controls (*p* < 0.05). CBT TV was lower than controls in both hemispheres for patients with NGT <6 mos, and lower for both hemispheres for patients with NGT > 6 mos. TV of the CBT in the affected hemisphere was negatively correlated with length of time until NGT removal in <6 mos group (r = 0.430, *p* < 0.05). NGT removed within 6 mos for individuals with unilateral but not bilateral injuries.	CD blinding; did not account for confounding variables in statistical analyses; no sample size justification
Lee et al. (2020) Retrospective cohort	Stroke: acute ischemic, VFSS	DWI; FLAIR Fazekas grade	VFSS	Clinical dysphagia scale	Corona radiata/internal capsule/basal ganglia	Bilateral lesions at the corona radiata/internal capsule/basal ganglia were significant prognostic factors for persistent dysphagia (*p* < 0.001).	CD blinding; no sample size justification; no details on white matter or swallowing analysis methods; collapsing brain areas into one category
Ko et al. (2019) Case control	Stroke: first unilateral involving corona radiata, CBT	NR Lesion size, Fazekas grade	Clinical swallow, VFSS for a portion	Feeding method, clinical judgement, dysphagia scale, PAS, NOMS, bolus timing	Corticobulbar tract	LA involving contralateral CBT was a significant predictor of feeding method at discharge (b = −3.95, OR = 0.02, *p* < 0.01) and NOMS score (b = 1.56, *p* = 0.03).	Limited information on WM assessment, Age was a confounding factor, no sample size justification, inconsistent use of video swallowing
Wilmskoetter et al. (2019) Retrospective cohort	Stroke: first hemispheric of the MCA	DWI VLSM ROI	VFSS	MBSIMP^©TM^ and PAS	Corona radiata, sup. longitudinal fasciculus, external capsule, ansa lenticularis, lenticular fasciculus	Regions surviving corrected threshold (z < −2.78) for impaired laryngeal elevation: Right external capsule (8.2%), right superior longitudinal fasciculus (0.1%), right superior corona radiata (0.2%); laryngeal vestibule closure (z < −3.43): right superior corona radiata (0.1%), right external capsule (5.5%); pharyngeal residue (z < −3.33): right superior corona radiata (1.2%), right posterior corona radiata (12.5%), right tapatum (1.7%), posterior limb of right internal capsule (0.2%), retrolenticular part of right internal capsule (3.9%), right superior longitudinal fasciculus (8.8%); PAS (z < −4.36): right superior longitudinal fasciculus (1.7%).	Statistical power higher in some brain regions (more damaged areas), missing data, limited discussion of power, CD blinding of outcome assessors
Fandler et al. (2018) Retrospective cohort	Stroke: recent small subcortical infarct	Lesion probability	GUSS	SLP GUSS rating: absent, mild, moderate, severe	Pyramidal tract, contralateral lacune, contralateral WM hypersensitivities (unspecified)	All patients with moderate or severe dysphagia had damage along the pyramidal tract, compared to 86% of patients without dysphagia. Patients with moderate to severe dysphagia more frequently had damage to the pyramidal tract and a contralateral pyramidal tract lacune (77.8 vs. 19.9%, *p* < 0.001).	Limited details on WM analysis, used a screener to identify dysphagia, did not incorporate confounds in stats, no sample size justification
Fandler et al. (2017) Retrospective cohort	Stroke: recent small subcortical infarct	MRI, DWI Lesion identification Fazekas grade	GUSS	SLP GUSS rating: absent, mild, moderate, severe	Unspecified	More severe WM hyperintensities were an independent predictor of dysphagia *(p* < 0.03), but that association was lost when analysis was restricted to patients with supratentorial damage (*p* < 0.27).	Screener for dysphagia, unspecified WM locations, no sample size justification, CD blinding for swallowing
Flowers et al. (2017) Retrospective cohort	Stroke	MRI, DWI ROI Fazekas grade	Clinical instrumental, or feeding method	NR	Internal capsule,	Internal capsule had an OR = 2.9 for dysphagia.	Did not give assessment details for swallowing, no sample size justification, heterogeneous sample, broadly defined regions, CD blinding
Galovic et al. (2017) Cohort	Stroke: first hemispheric, impaired oral intake	MRI, DWI VLSM ROI Tractography	Clinical Swallow. FEES if results “indeterminate”	Clinical Swallow (50 mL swallow test, Any 2 Scale, Gugging), FOIS	Sup. Corona Radiata, sup. longitudinal fascicle, external capsule, thalamic and cortico-bulbar projection fibers, fibers to contralateral thalamus	Statistical map of voxels associated with impaired oral intake after 7 days affected 89% WM with a center of maximum overlap over superior corona radiata with location immediately anterior to facial fibers (65% superior corona radiata, 12% superior longitudinal fascicle, 8% external capsule). Proportion of damaged voxels in the superior corona radiata was negatively correlated with degree of oral intake after 7 days (*p* = 0.001). After 4 weeks, the statistical lesion map covered 76% gray matter.	No classification of stroke severity, telephone assessment for last phase, no sample size justification
Jang et al. (2017) Single case study	Stroke	DWI ROI Tractography	NR	Severe dysphagia, fed by Levin tube	Corticobulbar tract	At the 5-week follow-up, the right CBT was discontinued at the subcortical right matter (Severe narrowing), left was not reconstructed. Right CBT recovered after rehabilitation and cranioplasty: thickened and extended to cerebral cortex. Resolution of dysphagia.	No details on swallowing measurement, limited demographic details, no discussion of power, no statistical analysis, CD blinding
Moon et al. (2017) Retrospective cohort	Stroke: mild first stroke, dysphagia symptoms, VFSS	MRI, DWI Fazekas grade	VFSS	Clinician description, bolus timing, penetration, aspiration	Unspecified	WM lesions are correlated with prolonged oral transit time (*r* = 0.384, *p* = 0.003) and increased penetration (*r* = 0.322, *p* = 0.015), even controlling for confounding variables. Mean oral transit time (OR = 3.082, *p* = 0.03) and penetration (OR = 2.521, *p* = 0.015) were significantly different in the severe WM lesion group than in the mild group. Left lesions associated with mastication (*p* = 0.039).	Unspecified WM lesions, subjective measures, no specific hypotheses, limited validated swallowing outcomes, no reliability testing, no sample size justification, CD blinding for swallow assessment
Mouräo et al. (2017) Cohort	Unilateral spastic cerebral palsy	MRI, DWI, FA, RD, MD, FC	Clinical Swallow	DDS, DMSS	Anterior, middle, posterior corpus callosum	Left hemisphere group (less severe): As FA (*r* = −0.667, *p* = 0.013) and FC decreased (*r* = −0.829, *p* < 0.001) and RD increased (*r* = 0.594, *p* = 0.032) (i.e., reduced structural integrity of the corpus callosum), dysphagia increased. Reduced FC in middle (*r* = −0.762, *p* = 0.002) and posterior (*r* = −0.739, *p* = 0.004) CC was associated with increased (worse) DDS. Right hemisphere group was more severe, and no significant correlations were observed.	Heterogeneous groups did not account for confounding variables in statistical analyses, no sample size justification
Galovic et al. (2016) Cohort	Stroke: first hemispheric stroke, failed dysphagia screening	MRI VLSM	Clinical Swallow	BODS-2	Sup. corona radiata, external capsule, sup. longitudinal fascicle	Mildly impaired oral intake was correlated with a widespread gray and white-matter network shown by a statistical map that included the superior corona radiata (12%), the external capsule (10%) and the superior longitudinal fascicle (8%).	No discussion of blinding, no sample size justification, limited accounting for confounding variables
Mihai et al. (2016) Case control	Stroke: first ischemic (recovered from Severe dysphagia w/in 3 years)	fMRI, DWI FA Lesion size	Clinical Swallow and VFSS for a portion	BODS-2, Neurogenic Oral Dysphagia test, water swallowing test	Pyramidal track laterality (between tongue and posterior limb of internal capsule)	Overall laterality of fractional anisotropy differed between patients and controls [*t*_(32)_ = 3.21, *p* < 0.005]. Patients showed asymmetric laterality of the pyramidal tract between tongue area and posterior limb of internal capsule. The larger the lesion, the more asymmetric (*r* = −0.676, *p* < 0.001). Laterality index was positively associated with compliance, meaning less compliant patients were less symmetric (*r* = 0.65, *p* < 0.009).	Lesions were heterogeneous, not all patients had VFSS, they each had individualized therapy which varied, hard to control for compliance, CD blinding
Wan et al. (2016) Retrospective cohort	Stroke: basal ganglia and/or centrum semiovale	MRI NR	VFSS FEES	Bolus timing, residue, physiologic observation	Centrum semiovale in conjunction with basal ganglia	83% of 12 patients had dysphagia.	No hypotheses, Small heterogeneous sample, no sample size justification, no diagnosis levels of severity, no MRI information, minimal participant information, no blinding, no confounding variables in analysis
Suntrup et al. (2015) Cohort	Stroke: first, failed dysphagia screening, FEES	MRI, DWI Regional lesion analysis	FEES	FEDSS ranking 1-6	Sup. longitudinal fasciculus (right and right temporal part), corticospinal tract (right)	Patients with dysphagia had a significant difference of mean percentage lesioned volume in the following areas: superior longitudinal fasciculus (right; *p* < 0.021, OR = 4.52), superior longitudinal fasciculus temporal part (right; *p* < 0.028, OR = 4.17), corticospinal tract (right; *p* < 0.044, OR = 2.79).	No sample size justification, limited methodology for WM and swallowing measures, unclear MRI settings, no confounding variables in analysis
Kim et al. (2014) Retrospective cohort	Stroke: first unilateral ischemic w/in 3 mos, dysphagia symptoms, VFSS	MRI Vascular white matter territory	VFSS	Physiologic observation, bolus timing, residue, penetration, aspiration	Unspecified	Excessive vallecular residue observed most frequently in the WM group (*p* < 0.002).	Unspecified WM lesions, no participant information, no assessment of stroke severity, no MRI specifications, no sample size justification, no confounding variables in analyses. CD blinding
Li et al. (2014) Cohort	Stroke: first hemispheric stroke of MCA	fMRI, DWI ROI, FA, seed-based connectivity	Clinical Swallow and VFSS	Logemann's indicators, PAS	Bilateral Corticospinal tract, Corpus Callosum	Reduced FA for left SMA to right SMA (corpus callosum), left SMA to internal capsule (corticospinal tract), and right SMA to internal capsule (corticospinal) in pts with dysphagia compared to controls. There were clear differences between stroke patients with dysphagia and healthy controls, but not between patients with and without dysphagia.	Limited detail on MRI, No sample size justification, no stroke severity, CD blinding, no confounding variables in statistical analyses, placed seeds for WM tracking close to cortex
Galovic et al. (2013) Prospective cohort	Stroke: first	MRI, DWI ROI lesion mapping	Clinical Swallow	Daniels et al. (1996) aspiration risk scale and BODS-2	Internal capsule, PVWM	Acute findings: internal capsule (OR = 7.6, *p* < 0.001), PVWM (OR = 4.8, *p* < 0.001). When adjusted for NIHSS and lesion size, internal capsule (OR = 6.2, *p* < 0.002) and now PVWM OR = 2.7, *p* < 0.06). Model accuracy 76%. No WM areas were associated with extended risk of aspiration.	No sample size justification, CD blinding, Clinical “risk of aspiration” with no instrumental, no mild strokes, only analyzed early subacute phase
Kumar et al. (2012) Retrospective cohort	Stroke: ischemic, severe dysphagia	DWI Lesion volume	Clinical and/or video swallow	Severe dysphagia = absence of oral intake or significant aspiration	Unspecified	In univariate analysis PVWM was not a significant predictor of PEG placement. In a multivariate analysis with age, NIHSS score, lesion volume, and brain locations, PVWM approached significance (*p* <0.057 with OR = 3.829). NIHSS score and Bihemispheric lesions were significant predictors.	Unspecified white matter lesions, only participants with severe dysphagia, limited details on measurement methods, no sample size justification, CD blinding
Cola et al. (2010) Case control	Stroke: unilateral ischemic subcortical	DWI Lesion volume	VFSS	Bolus timing, PAS, bolus clearance	Unspecified	Significant interaction between peri-ventricular WM lesions and hemisphere (chi square = 9.85, *p* = 0.002). 100% had dysphagia in LH PVWM group, 0% dysphagia for RHD. No association was detected for those without PVWM damage.	Unspecified WM lesions, Small n, no MRI in healthy subjects, controls were not matched for race, no sample size justification
Levine et al. (1992) Cohort	n/a	MRI MRI score	VFSS	Bolus timing	Unspecified	Total swallow duration (*p* < 0.009) and oral transit duration (*p* < 0.047) significantly differed by MRI score (total number of WM unidentified bright objects).	Unspecified WM lesions, only healthy controls, no sample size justification, limited participant details, old imaging methods

*Order of appearance: NGT, nasogastric tube; DWI, diffusion weighted imaging; FA, fractional anisotropy; TV, tract volume; VFSS, videofluoroscopic swallow study; PAS, penetration aspiration scale; FOIS, functional oral impact scale; CBT, corticobulbar tract; CD, cannot determine; GUSS, Gugging Swallow Screen; LA, leukoaraiosis (hyperintensity around ventricles); ASHA, NOMS American Speech Language Hearing Association National Outcome Measurement System; OTT, Oral Transit Time; PTT, Pharyngeal Transit Time; OR, odds ratio; WM, white matter; MCA, middle cerebral artery; NIHSS, national institute of health stroke scale; VLSM, voxel based lesion symptom mapping; ROI, regions of interest; MBSIMP^TM^, Modified Barium Swallow Impairment Profile; Sup, Superior CNS Canadian Neurological Scale; MRI, magnetic resonance imaging; SLP, speech language pathologist; NR, Not Rated; FEES, Fiberoptic Endoscopic Evaluation of Swallowing; GMFCS, gross motor function classification scale; MACS, manual ability classification scale; RD, radial diffusivity; MD, mean diffusivity; FC, fibers count; DDS, Dysphagia Disorder Survey; DMSS, dysphagia management staging scale; fMRI, functional magnetic resonance imaging; BODS-2, Bogenhausen Dysphagia Score Part 2; FEDSS, fiberoptic endoscopic dysphagia severity scale; SMA, supplementary motor area; PVWM, periventricular white matter*.

Eight studies used a clinical (bedside) swallowing evaluation (CSE), which typically includes a case history, a detailed cranial nerve assessment, oropharyngeal mechanism exam, and oral trials of foods and liquids. One study used a CSE in addition to VFSS (Li et al., 2014), four studies used a CSE for all patients, while reporting that a portion of subjects also received instrumental assessments (Kumar et al., 2012; Mihai et al., 2016; Galovic et al., 2017; Ko et al., 2019), and three studies used only a CSE (Galovic et al., 2013, 2016; Mouräo et al., 2017). Lastly, in two studies the researchers performed only a swallow screening, i.e., a brief evaluation determining the risk for a diagnosis of dysphagia (Fandler et al., 2017, 2018).

#### Analysis of Swallowing Parameters

Table 4 also summarizes each study's swallowing analysis methods (see Supplementary Table C for more extensive detail). Of the studies that included VFSS for all subjects (*n* = 9), six used the Penetration Aspiration Scale (PAS) which rates the level of airway invasion and patients' response to penetration or aspiration events (Rosenbek et al., 1996), five studies employed temporal/timing measures, and Wilmskoetter et al. (2019) used the Modified Barium Swallow Impairment Profile (MBSImP^©™^; Martin-Harris et al., 2008), a standardized protocol that enables clinicians to quantify physiological swallowing impairments. Of the studies that used FEES, one study used a tool to rate dysphagia severity from FEES, the fiberoptic endoscopic dysphagia severity scale (FEDSS).

Five of the eight studies that included CSEs used standardized tools to interpret the assessment. Mouräo et al. (2017) used the Dysphagia Disorder Survey, a validated clinical assessment of swallowing and feeding function for individuals with intellectual and developmental disability (Sheppard et al., 2014); three studies used the Bogenhausen Dysphagia Score, Part 2 (BODS-2) (Bartolome, 2006), which is a German assessment of oral intake; and two used the Functional Oral Intake Scale (FOIS) (Crary et al., 2005), i.e., a description of levels of oral intake, retrospectively. Three studies reported mixed methods for swallowing analysis, such as a variety of different clinical scales (see details on all methods in Supplementary Table C).

### White Matter Areas Implicated in the Neural Control of Swallowing

#### Quality of Evidence and Bias Assessment

In order to determine if there is definitive evidence implicating specific white matter areas in the control of swallowing (Research Question 3), we first critically assessed the quality of the available evidence using the modified NIH quality assessment (results in last column of Table 1). Five articles were rated as “Good,” fifteen articles were classified as “Fair,” and two articles were classified as “Poor.”

The most common risk of bias (found in 20 of the 21 studies with greater than one participant) involved failing to justify sample size (i.e., not reporting power analysis or variance/effect estimates) (Table 1). Sample size was frequently limited by the clinical setting and/or by the retrospective design. The second most frequent item impacting quality assessment was blinding. Twenty of the 22 studies did not describe blinding of assessors (e.g., whether swallowing assessors were blinded to MRI results/diagnoses). Other elements negatively affecting quality ratings were related to the measurement methods used to evaluate swallowing and/or white matter. For example, studies frequently included the use of a non-validated and/or non-standardized swallowing assessment tool (15 of 22 studies) or reported limited details on MRI methodology/imaging. Lastly, for 13 studies, ratings were affected by not including confounding variables in statistical analysis. Although handling of missing data was not quantified by the NIH quality assessment tool we used, we noted that few studies described whether they had missing data and how they handled it.

Robust statistical analysis was a common element among all papers that received a quality rating of “Good.” Although only five papers received this highest rating, 20 of 22 papers were of at least “Fair” quality. Therefore, to answer Research Question 3 we qualitatively synthesized the findings of these 20 studies. The two studies rated as “Poor” were not included in this synthesis, due to significant risk of bias, but their information is presented in the tables for the sake of completeness.

#### Specific White Matter Tracts of Interest and Their Roles

Six of the twenty “Good” or “Fair” quality studies discussed “periventricular white matter” without further location specificity, and fourteen provided information on specific white matter regions implicated in swallowing control (Table 4; see Supplementary Table C for more detailed summary), albeit with differing levels of specificity. The most commonly implicated white matter tracts across studies were the pyramidal tracts (*n* = 8), followed by more specific tract sections, such as the internal capsule (*n* = 4), the superior longitudinal fasciculus (*n* = 3), the corona radiata (*n* = 3), the corpus callosum (*n* = 2), the external capsule (*n* = 2), and the ansa lenticularis/lenticular fasciculus (*n* = 1).

##### Pyramidal tracts and subdivisions

Eight studies identified the pyramidal tracts as important in swallowing control. The pyramidal tracts are the projection fibers carrying motor information from the cortex to the brainstem and spinal cord. Although these fibers are frequently subdivided into the corticobulbar and corticospinal tracts (Lohia and McKenzie, 2020a), two studies referred to the pyramidal tract as a whole. Specifically, Fandler et al. (2018) examined 243 patients with dysphagia and found that all patients (100%) with moderate or severe dysphagia had damage along the pyramidal tracts, compared to 86% of patients without dysphagia (Fandler et al., 2018). Further, patients with moderate to severe dysphagia more frequently presented with damage to one pyramidal tract (left or right) *and* a white matter hyperintensity on the contralateral pyramidal tract than those without dysphagia (77.8 vs. 19.9% respectively, *p* < 0.001). In a study by Mihai et al. (2016), both clinical swallowing assessments and task-based fMRI and DWI scans were performed in 18 patients who had recovered from clinically determined post-stroke dysphagia and 18 healthy controls. Results of the DWI-based FA analysis revealed that, in comparison with the control group, the patient group had an asymmetric laterality index of the pyramidal tract FA with reduced FA mostly ipsilesionally. This indicated involvement of pyramidal tract lesions in the development of dysphagia, but also some neuroplastic capacity that played a role in recovery.

Two additional studies implicated one division of the pyramidal tract, the corticospinal tract. Li et al. (2014) found that stroke patients with dysphagia had reduced FA in the corticospinal tracts bilaterally when compared to healthy controls, but their FA was not statistically different than stroke patients without dysphagia. This finding may have been influenced by the small sample size (*n* = 12 in each group) (Li et al., 2014). In a larger study (*n* = 200), patients with damage to the right corticospinal tract had significantly higher odds of being diagnosed with dysphagia than patients without damage to the same area (OR = 2.79, *p* < 0.044; Suntrup et al., 2015).

The other division of the pyramidal tract, the corticobulbar tract (CBT), is critical for bulbar functions such as swallowing and speech and was implicated in four papers. Three of these papers directly aimed to investigate the contribution of the CBT in swallowing control. Two studies evaluated the predictive value of CBT damage on prognosis for dysphagia recovery (Jang et al., 2020a,b). Specifically, Jang et al. (2020a) used tractography to measure FA and TV of the CBT in 42 patients with intracerebral hemorrhage and subsequent dysphagia requiring nasogastric tube (NGT) placement. Patients who recovered swallowing within 2 days had relatively minor damage to the CBT (only reduced FA, not reduced TV), whereas patients with longer NGT placement had more extensive damage to the CBT (both reduced FA and TV). In patients with longer NGT placement (2 days to 6 months), CBT volume in the affected hemisphere was negatively correlated (*r* = −0.430, *p* < 0.05) with length of time until NGT removal. Finally, none of the patients who had bilateral damage to the CBT were able to have their NGT removed within 6 months (Jang et al., 2020a). In a separate study using similar methodology, Jang et al. (2020b) examined 20 patients with lateral medullary infarctions and found that CBT FA was significantly lower in patients with prolonged NGT placement (<6 months) compared to controls and to patients with shorter NGT placement durations (Jang et al., 2020b, p. 20). Ko et al. (2019) examined the impact of unilateral vs. bilateral damage to the corticobulbar tract (CBT) on swallowing (Ko et al., 2019). They investigated two groups of stroke patients with lesions involving the CBT: one group with unilateral CBT damage and one group with bilateral CBT involvement [defined as damage to the CBT in one hemisphere and *additional* CBT leukoaraiosis (i.e., white matter hyperintensity) contralaterally]. As expected, bilateral CBT involvement independently predicted worse performance on functional swallowing measures (Ko et al., 2019).

Finally, the involvement of the CBT in swallowing was also reported in a single case study including a stroke patient with damage to the middle cerebral artery and subsequent intracerebral hemorrhage (Jang et al., 2017). At 5 weeks post-stroke, the patient was reported to exhibit severe dysphagia and extensive brain swelling, accompanied by a severely narrowed right CBT, which was not extending to the cortex, and no identifiable left CBT. After decompressive craniotomy at 8 weeks post-stroke, dysphagia symptoms resolved and imaging showed decreased swelling, and a more “normal appearing” right CBT that now extended to the cortex. This indirectly suggests the role of CBT fibers in connecting areas of the swallowing network.

##### Internal capsule

The internal capsule is a white matter structure which contains both ascending (i.e., thalamocortical) and descending (i.e., pyramidal) fibers, and therefore carries both sensory and motor information. Damage to the internal capsule was associated with dysphagia or aspiration risk in four studies. Flowers et al. (2017) retrospectively reviewed 160 stroke patients to examine neuroanatomical factors that predict the diagnosis of dysphagia, aphasia, and/or dysarthria. They identified seventy-six patients with post stroke dysphagia, and they reported that damage to the internal capsule increased odds of being diagnosed with dysphagia by an average of 3 times (OR = 2.9; 95% CI 1.2–6.6) (Flowers et al., 2017). Galovic et al. (2013) examined lesion location as a predictor of aspiration risk [assessed using the Daniels' et al. clinical evaluation method (Daniels et al., 2000)] in 94 patients within 48 h post stroke and at ~1-week post-stroke. They found that patients with internal capsule lesions had increased odds of aspiration risk in the acute phase (OR = 4.8, *p* < 0.001), but not at 1-week post-stroke [OR = 1.3, *p* = 1.0; (Galovic et al., 2013; Flowers et al., 2017)]. Further evidence for the involvement of the internal capsule in swallowing control derives from Mihai et al. DWI-based FA analysis (Mihai et al., 2016). Similar to the results involving the pyramidal tracts, their patient group had an asymmetric laterality index of the posterior limb of the internal capsule FA with reduced FA ipsilesionally, also suggesting involvement of this specific white matter area in the recovery of swallowing function. Lastly, Lee et al. (2020) found that bilateral internal capsule/corona radiata/basal ganglia lesions (all grouped together) were significant prognosticators for persistent dysphagia (*p* < 0.001) (Lee et al., 2020).

##### Superior longitudinal fasciculus

The superior longitudinal fasciculus, an association tract, connects multiple brain regions including the frontal, occipital, parietal, and temporal lobes, creating the networks needed for the regulation of motor behavior and conveyance of somatosensory information. Involvement of the superior longitudinal fasciculus was reported in three studies. In two of these, the Galovic group used VLSM to examine lesion locations and connectivity patterns as predictors of impaired oral intake in the acute stroke phase (~2 days post-stroke) (Galovic et al., 2016) and at ~1 and 4 weeks post stroke (Galovic et al., 2017). Their results showed that the statistical map of voxels associated with impaired oral intake involved the superior longitudinal fasciculus to a small extent (8 and 12% of voxels), in the acute and ~1-week phases, respectively (Galovic et al., 2016, 2017). Further, Suntrup et al. (2015) also used voxel-based imaging analysis to examine whether stroke location is associated with dysphagia in 200 acute stroke patients. They reported that damage to the right superior longitudinal fasciculus or the temporal part of the right superior longitudinal fasciculus increased the odds of dysphagia diagnosis by ~4 times. This tract was also reported in a more recent retrospective study that investigated the association between lesion location (using VLSM) and physiological aspects of swallowing (rated using the MBSIMP^©TM^ and the PAS) in 68 acute stroke patients (Wilmskoetter et al., 2019). After controlling for age, time between measurements, and lesion volume, this study found that lesions including the superior longitudinal fasciculus were associated (to a small extent) with impairment in three physiological components. Specifically, the superior longitudinal fasciculus was implicated in 1.7% of lesioned voxels associated with increased PAS scores, 8.8% of voxels associated with pharyngeal residue, and 0.1% of voxels associated with impaired laryngeal elevation (Wilmskoetter et al., 2019).

##### Corona radiata

The corona radiata is a collection of both ascending and descending white matter tracts that spread toward the cortex and connect with the internal capsule. Three studies found that damage to the corona radiata was associated with some swallowing deficits. Galovic et al. reported two interesting findings regarding this region in their 2017 study examining the associations between lesion locations and impaired oral intake at ~1 and 4 weeks post stroke. First, they found that at ~1-week post-stroke, the statistical map of voxels associated with impaired oral intake included lesions in the superior corona radiata at a greater extent than any other area (65% of lesioned voxels). Secondly, at the same time point, the percent of damage in this area was negatively correlated with the degree of oral intake and the majority of patients with lesions in more >50% of the corona radiata had impaired oral intake (Galovic et al., 2017). In the 2016 study by the same research group, the corona radiata was identified in 12% of lesioned voxels associated with decreased oral intake at 48 h post-stroke (Galovic et al., 2016), further implicating this tract in swallowing control. The Wilmskoetter et al. study (2019) also reported that lesions including the right superior corona radiata were associated to a small extent with impaired laryngeal elevation (voxel overlap of 0.2%), impaired laryngeal vestibular closure (voxel overlap of 0.1%), and pharyngeal residue (voxel overlap of 1.2%). Lesions including the right posterior corona radiata were associated to a slightly larger extent with pharyngeal residue scores (voxel overlap of 8.8%) (Wilmskoetter et al., 2019). Finally, as reported previously, Lee et al. (2020) found that bilateral internal capsule/corona radiata/basal ganglia lesions (all grouped together) were prognosticated persistent dysphagia (Lee et al., 2020).

##### Corpus callosum

The corpus callosum (CC) was identified in two papers as important in swallowing control. In children with CP and left hemisphere lesions affecting primarily the sensorimotor cortex area (*n* = 13), increased clinical signs of dysphagia were correlated with reduced structural integrity of the corpus callosum quantified by FA decrease (*r* = −0.667, *p* = 0.013), fiber count decrease (*r* = −0.829, *p* < 0.001), and radial diffusivity increase (*r* = 0.594, *p* = 0.032) (Mouräo et al., 2017). In particular, reduced fiber count in the middle (*r* = −0.762, *p* = 0.002) and posterior (*r* = −0.739, *p* = 0.004) corpus callosum was associated with increased (worse) total score on the Dysphagia Disorder Survey for this group of children. A similar pattern was not observed for the group of children with right hemisphere lesions (*n* = 7), however the vast majority of these children had subcortical or peri-ventricular white matter (PVWM) lesions affecting intra-hemispheric connections. The authors concluded that CC integrity and inter-hemispheric communication might be more critical for swallowing control when the sensorimotor cortex is impacted, and not as critical when subcortical intra-hemispheric connections are disrupted (Mouräo et al., 2017). In the study by Li et al. (2014) including stroke patients with and without dysphagia and healthy controls, mean FA for the corpus callosum was significantly decreased in stroke patients with dysphagia when compared to the healthy controls, but when these stroke patients were compared to the group of patients without dysphagia, this difference was not significant.

##### External capsule

The external capsule, a series of association tracts between the putamen and claustrum, was reported in three studies. In the 2016 Galovic et al. study, 10% of lesioned voxels associated with impaired oral intake at the acute stroke phase (<48 h after imaging) overlapped the external capsule (Galovic et al., 2016). In the 2017 study by the same group, 8% of lesioned voxels associated with impaired oral intake at 1-week post-stroke overlapped this white matter area (Galovic et al., 2017). Finally, Wilmskoetter et al. (2019) reported that damage to the right external capsule was associated to some extent with impaired laryngeal elevation (voxel overlap of 8.2%) and impaired laryngeal vestibule closure (voxel overlap of 5.5%) in their sample of 68 patients post stroke (Wilmskoetter et al., 2019).

### Three Additional Themes

In addition to insights on specific white matter tracts, there were several studies that generally investigated white matter and its role in swallowing control, without specifying tract locations. Three themes emerged from this literature and were reinforced by some previously discussed studies. These were topics on lesion severity, hemispheric involvement, and time post-stroke.

#### Lesion Severity

Although lesion severity was not consistently reported in all studies included in this synthesis, six studies indicated that severity of the white matter lesion impacts components of swallowing. In a retrospective study of 63 mild stroke patients (NIHSS ≤ 5), severity of white matter lesions (measured using the Fazekas scale) was correlated with prolonged oral transit time (*r* = 0.384, *p* = 0.003) and increased penetration occurrences (*r* = 0.322, *p* = 0.015), even after controlling for variables such as age, sex, initial stroke severity, lesion laterality, and lesion location (Moon et al., 2017). In addition, a larger retrospective study including 322 stroke patients found that a higher NIHSS score (indicating higher stroke severity) and more severe white matter hyperintensities identified in MRI scans were both identified as risk factors for suspected dysphagia as measured with the Gugging Swallow Screen. However, when the analysis was restricted to patients with supratentorial damage, white matter hyperintensities did not remain significant risk factors (Fandler et al., 2017). Further, severity of damage to one specific white matter tract, the CBT, predicted prognosis for dysphagia recovery in two studies by Jang et al. (2020a,b).

In another study, by Kumar et al. (2012), the aim was to examine the influence of age, NIHSS score, time post stroke, and lesion characteristics in predicting placement of a percutaneous endoscopic gastrostomy (PEG) tube in 77 patients with severe dysphagia resulting from an acute-subacute hemispheric lesion. Baseline NIHSS score and bilateral hemispheric involvement were the most significant predictors of PEG tube placement in this cohort (Kumar et al., 2012). Further, as reported earlier, in the study by Galovic et al. (2017) the amount of damage in the superior corona radiata was negatively correlated with the degree of oral intake, further implicating that white matter lesion load or severity plays a role in the development of swallowing difficulties (Galovic et al., 2017).

Finally, in a prospective study of 49 healthy adults (43 to 79 years of age), VFSS evaluations and a brain MRI scan were performed in order to examine the effect of subtle changes to white matter, or “unidentified bright objects,” on temporal/durational aspects of swallowing. Results showed that total swallow duration (*p* < 0.009) and oral transit duration (*p* < 0.047) differed significantly by MRI score, i.e., by number of “unidentified bright objects” in white matter (Levine et al., 1992). This was the only study included in this review that indicated that even in healthy individuals, small changes/aberrations to white matter might be influential for swallowing control.

#### Hemispheric Involvement

Three studies found a potential effect of lateralization of a white matter lesion to dysphagia diagnosis and/or severity. Cola et al. (2010) investigated 20 acute stroke patients, ten with left subcortical damage and ten with right subcortical damage. They found a significant statistical interaction between hemisphere and lesion location (chi square = 9.85, *p* = 0.002) and concluded that lesions to the left PVWM may be more disruptive to swallowing that right PVWM lesions. On the other hand, the study by Wilmskoetter et al. (2019) found that the majority of gray and white matter areas implicated in swallowing dysfunction in their post-stroke sample were in the right hemisphere. However, they also observed that two of four pharyngeal components of the MBSIMP^©TM^ were associated with some lesions to the left hemisphere, thus concluding that although both hemispheres play a role in swallowing control, the control of the right hemisphere appears to be more prominent (Wilmskoetter et al., 2019). Similarly, the study by Mouräo et al. (2017) reported that children with CP and right hemisphere lesions (the majority of which were in the PVWM area) presented with more severe clinical dysphagia compared to children with CP with left hemisphere lesions (Mouräo et al., 2017). However, the majority of children in the left hemisphere group did not have PVWM lesions and there was a relatively small sample of children in each subgroup (13 left hemisphere, 7 right hemisphere), limiting the interpretation of this finding. Regardless of the individual contributions of left and right hemispheres, there is evidence that bilateral lesions, particularly to the pyramidal tract, tend to be more disruptive than unilateral lesions (Kumar et al., 2012; Ko et al., 2019; Jang et al., 2020a,b).

#### Time Post-stroke

Additionally, two studies demonstrated that white matter damage may have particular clinical relevance in the acute post-stroke phase. Galovic et al. (2017) reported that the map of lesioned areas associated with impaired oral intake at 1-week post-stroke affected white matter structures in 89% (of the voxels), whereas the respective map for patients with persistent dysphagia at 4 weeks post stroke covered mostly gray matter areas, and only 24% white matter (Galovic et al., 2017). Similarly, the same research team previously (2013) reported that patients with damage to PVWM had higher aspiration risk at 48 h post-stroke compared to patients without PVWM damage (OR = 4.8, *p* < 0.001) (Galovic et al., 2013). Both studies indicate that disruptions in white matter areas early post-stroke likely disrupt the communication between gray matter areas that are critical in swallowing, but also that recovery of these connections can occur quickly and can be essential in helping restore swallowing function.

### Gaps in the Investigation of White Matter and Swallowing

The last research question (Research Question 4) sought to identify specific gaps in the literature in order to help guide future research in this area. Through our qualitative synthesis three major gaps were identified. These gaps were: (1) limited representation of populations, (2) imaging and swallowing methodology, and (3) research design and statistical rigor.

#### Limited Representation of Populations

Two clinical populations were represented in the available literature: patients post stroke (20 studies) and children with cerebral palsy (1 study), along with one study examining variability within healthy adults (Levine et al., 1992). Representation across age, race, and ethnicity was significantly limited (Table 2). Only one study included pediatric patients; all other studies included wide adult age ranges, often without accounting for effects of age in their statistical analysis. Finally, only two studies reported race, and one reported ethnicity.

#### Imaging and Swallowing Methodology

Studies included in this review greatly varied in both imaging and swallowing methodology used, and critically few used current gold standard methodology for both measures. Specifically, most identified articles measured white matter lesions and rarely used methods to measure white matter integrity, such as tractography, fractional anisotropy, radial diffusivity, mean diffusivity, and fibers count. There were also inconsistences in the reporting of imaging parameters (such as signal strength or scanner type), which limits interpretation of findings. However, clinical scales that were reported across multiple studies, such as the Fazekas scale, improved comparability between those studies. In regard to swallowing evaluation and analysis, relatively few papers used gold-standard swallowing measurement methods (VFSS or FEES) in conjunction with validated analysis tools (Table 4). Even when VFSS or FEES methodologies were utilized, the measures used to analyze the data were often limited or not validated (Table 4).

#### Research Design and Statistical Rigor

Finally, issues with research design or statistical rigor were observed across many studies (see Tables 1, 4). Several retrospective studies included inconsistent assessment methods or lacked carefully controlled research protocols. Further, few studies provided evidence of adequate power or reported blinding outcome assessors, and less than half of the studies thoroughly controlled for the potential impact of confounding variables in their statistical analysis. One key area that should also be highlighted in future studies is incorporation of confounding factors in research design and analysis.

These identified gaps in representation, methodology, and study design are important and need to be carefully considered in future research.

## Discussion

The majority of swallowing neurophysiology work has focused on the contributions of CNS gray matter in the control of human swallowing, and much less attention has been given to the white matter tracts that form connections between gray matter areas. These tracts hold promise for patients with dysphagia because, in addition to being communication highways, they are also known to be primary drivers of recovery after injury or disease and are highly capable of adaptation with rehabilitation and re-learning (Trivedi et al., 2008; Schulz et al., 2014; Kato and Izumiyama, 2017, p. 201; Barghi et al., 2018).

In this systematic review, we sought to identify and systematically evaluate the literature describing the role of white matter in the neural control of swallowing in order to answer four primary questions: what patient populations have been studied in this literature; what methodologies have been used to assess white matter integrity and swallowing; what specific white matter tracts are implicated in swallowing control; and what are the main gaps in the literature that need to be addressed in future research.

To summarize, we identified 22 articles that fit our inclusion criteria. All studies were observational, i.e., there was no intervention assessed, and almost half followed a retrospective cohort design, with the other half being either case control studies or variations of prospective cohort designs. Using a modified NIH quality assessment protocol (Study Quality Assessment Tools | NHLBI, NIH, 2019) (Table 1), five studies were rated as having “Good” quality, 15 studies were of “Fair” quality; and two studies were rated as “Poor” and were excluded from the qualitative synthesis used to answer question 3 of this review.

Regarding the first research question (populations), stroke was by far the most represented diagnosis in the identified literature. Only two of the 22 reviewed papers examined different populations; one study examined children with CP, and one focused on healthy older adults. The majority of studies included *acute* adult stroke patients, with almost exclusively first strokes with no prior infarcts or comorbidities, and a mix of types of strokes (see Table 4). This proportionally high representation of one diagnosis in the literature is likely due to relevance and convenience. Patients post stroke often exhibit white matter damage with subsequent deficits, and also have readily available neuroimaging scans that can be studied retrospectively. Since neuroimaging is costly, it is unsurprising that research on this topic has started with a population who has existing scans and related damage. However, white matter damage has been documented in other populations that are also at high risk for developing swallowing difficulties, including people with traumatic brain injury (Herrera et al., 2016), dementia (Love and Miners, 2015), chronic drug abuse (Narayana et al., 2014), multiple sclerosis (Tassorelli et al., 2008), and gestational hypoxia (Baud et al., 2004; Kaur and Ling, 2009), as well as in typical aging (Metzler-Baddeley et al., 2019). In addition to a gap in representation of clinical populations, few of the reviewed studies included healthy control participants or focused on white matter integrity measures in healthy participants, leaving an additional gap in our understanding of white matter connections in normal swallowing.

In regard to the second question (methodologies to assess white matter and swallowing), we observed an interesting dichotomy. The majority of studies that measured characteristics of the white matter structures themselves (e.g., FA, tract volume, mean diffusivity, etc.), evaluated swallowing *via* clinical methods or screenings, instead of using instrumental tools. On the other hand, most of the studies that examined swallowing physiology in some depth or with validated tools tended to utilize the more crude (or lesion-based) white matter imaging techniques (e.g., lesion volume calculations, lesion severity scales, etc.).

Imaging advancements in the use of DWI/DTI have allowed for a significant increase in our understanding of brain connections and their role in recovery and rehabilitation in related fields (Trivedi et al., 2008; Schlaug et al., 2009; Huber et al., 2018). For this reason, we expected to find these methodologies used in swallowing neurophysiology literature as well. However, we observed that very few of the reviewed studies utilized these sequences to investigate white matter integrity in their samples, despite the fact that many research groups had access to the relevant DWI scans. Instead, they relied on lesion-based methods, which are useful when examining patients with stroke or another pathology with specific lesions, but do not quantifiably measure white matter itself. Since lesions often affect multiple brain areas at once this approach can result in unspecified conclusions. Alternatively, techniques that measure and describe characteristics of white matter, such as FA, radial diffusivity, mean diffusivity, or fibers count, reveal changes specific to the white matter structures. These techniques are sensitive enough to detect differences even within healthy populations (Madden et al., 2008; Ziegler et al., 2010; Bennett et al., 2011; Schulz et al., 2014). The few studies in this review that used these techniques indicated that these metrics could be valid predictors for swallowing outcomes post stroke (Jang et al., 2020a,b), and they helped identify more specific white matter tracts of interest for swallowing control (Mouräo et al., 2017; Wilmskoetter et al., 2019).

To further delineate elements critical to advancing these efforts, we also reviewed these studies' swallowing methodology. Fifteen of the identified studies used gold-standard measures that allow in-depth evaluation of the oropharyngeal swallowing phases (VFSS or FEES). Further, several of these studies used timing/temporal measures to quantify imaging analysis, and one study used the MBSIMP^©TM^ to standardize clinical interpretation. This allowed for more in-depth discussion of subcomponents of swallowing in relation to nervous system damage. For the remaining studies that did not use instrumental methods, it is difficult to adequately characterize the underlying mechanisms of dysphagia in their participants. This was, in some cases, partially ameliorated with the use of validated and standardized clinical assessments. Our understanding of swallowing physiology in relation to white matter integrity could be improved through detailed kinematic or morphometric analysis methods (Molfenter and Steele, 2014; Pearson et al., 2016), which help assess aspects of swallowing physiology more objectively.

Our findings on question 2 highlight the importance of combining high quality neuroimaging and swallowing physiology expertise to comprehensively investigate the neural control of swallowing. The need for strong interdisciplinary collaborations that will enable the combination of recent advanced imaging methods with in-depth swallowing evaluation and analysis techniques is apparent.

**Figure 2:**
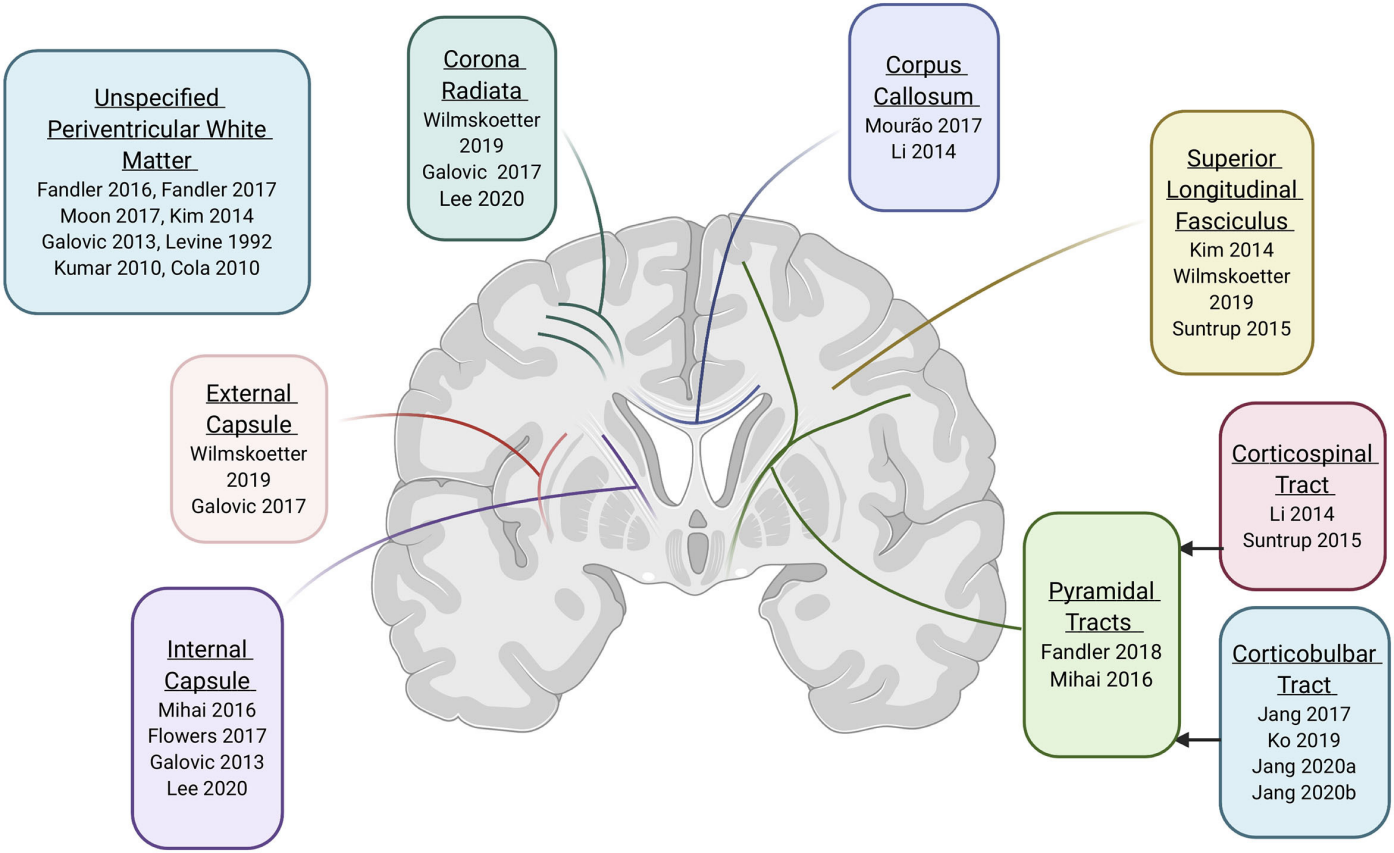
Evidence for specific white matter tracts involved in neural control of swallowing (created with Biorender.com).

Given the methodological issues described above, for question 3 (i.e., white matter tracts implicated in swallowing control), we completed a synthesis of the findings of 20/22 studies that had a quality rating of at least Fair. Since the methods and results of the studies reviewed were heterogeneous, we cannot definitively describe the specific role of white matter in the neural control of swallowing. However, through our synthesis, the following white matter tracts or structures were frequently reported (Figure 2): the pyramidal tract (as a whole) and some of its subparts like the corticospinal and corticobulbar tracts, the internal capsule, the superior longitudinal fasciculus, the corona radiata, the corpus callosum, and the external capsule.

Fibers in three of these tracts/structures are categorized as projection fibers. Projection fibers were implicated in four of five studies that received a “Good” quality rating. Projection fibers connect the cortex with deep nuclei, lower parts of the brain (i.e., the brainstem), and the spinal cord. The role of the cortex and brainstem are extensively documented in animal and human studies of the neural control of swallowing (e.g., Jean, 1984; Robbins et al., 1993; Hamdy et al., 1997; Malandraki et al., 2011), so it logically follows that disruptions in the connections between these areas could affect swallowing. Specifically, dysphagia was frequently associated with bilateral damage to the pyramidal tracts, internal capsule, or corona radiata. The pyramidal tract originates in the bilateral supplementary motor area and dorsal premotor area (Wang et al., 2019) and ends at the brainstem (corticobulbar tract) or spinal cord (corticospinal tract) (Lohia and McKenzie, 2020b), and was the most clearly implicated projection tract. This is unsurprising since the supplementary motor area (SMA) has been repeatedly implicated in volitional swallowing, particularly in the preparatory phase (Huckabee et al., 2003; Satow et al., 2004; Hamdy, 2006; Malandraki et al., 2009), and communication between the SMA and subcortical areas (e.g., the basal ganglia and brainstem) is needed for swallow initiation (Hamdy et al., 1996). A subpart of the pyramidal tract, the corticobulbar tract, was reported in five reviewed papers, with two papers finding that severity of CBT injury appears to have prognostic value for predicting swallowing recovery post-stroke (Jang et al., 2020a,b).

Fibers in the superior longitudinal fasciculus and external capsule are categorized as association fibers and connect brain regions within the same hemisphere. In two of the papers rated as “Good,” damage to these fibers was also associated with swallowing deficits. The superior longitudinal fasciculus carries input from parietal sensorimotor centers to frontal motor areas influential for swallowing coordination and initiation (Schmahmann et al., 2008). The external capsule connects the pre-frontal cortex and the supplementary motor area with the basal ganglia, and it has been hypothesized to be the key for the engagement of the basal ganglia in swallowing motor control (Schmahmann et al., 2008). Damage to these tracts was associated with impaired oral intake post-stroke and was associated (to a small extent) with deficits in specific pharyngeal subcomponents of swallowing in one study (Wilmskoetter et al., 2019).

Finally, one commissural tract, the corpus callosum, was identified in two studies. It is established that swallowing involves bilateral cerebral control (Hamdy et al., 1999; Malandraki et al., 2009), and the main pathway connecting the hemispheres is the corpus callosum. Although direct evidence is scarce, it has been theorized that the corpus callosum may be involved in communications between swallowing areas in the right and left hemispheres (Mouräo et al., 2017). In the Mouräo et al.' study (2017), which included a relatively small sample of children with CP (*n* = 20), it was concluded that disruptions in the corpus callosum were more influential for swallowing control when lesions affected cortical MCA areas, thus suggesting some influential disruptions in interhemispheric connections. Given that the quality of the studies implicating the corpus callosum was “Fair,” further research is needed to elucidate the role of inter-hemispheric connectivity for swallowing control.

In addition to insight on specific white matter tracts/structures of interest, there were three common themes regarding severity, hemispheric contribution, and time post-stroke that emerged from this literature. First, studies provided evidence that severity of white matter lesions was predictive of dysphagia severity and/or recovery (Jang et al., 2020a,b). Further, even among healthy individuals, changes to white matter were associated with changes in swallowing control (i.e., total swallow duration and oral transit duration) (Levine et al., 1992). This finding is consistent with prior literature, supporting that severity of impairment from stroke (NIHSS score) moderately predicts clinically relevant dysphagia (Jeyaseelan et al., 2015), and lesion severity also predicts post-stroke dysphagia (Otto et al., 2016; Cabib et al., 2017; Rofes et al., 2018) and warrants further investigation.

The second theme involved the role of each hemisphere's white matter in swallowing control. White matter lesions of both hemispheres were reported to correlate with swallowing deficits, but more evidence pointed to the potential influence of the right hemisphere's white matter areas (Mouräo et al., 2017; Wilmskoetter et al., 2019). This finding is not surprising, as several prior neuroimaging studies have also shown that gray matter areas of the right hemisphere play a more prominent role in the pharyngeal phase of swallowing compared to areas in the left hemisphere (Robbins et al., 1993; Daniels et al., 1996, p. 199; Hamdy et al., 1996; Malandraki et al., 2010; Wilmskoetter et al., 2018). In addition, there was consistent evidence that bilateral white matter damage is particularly disruptive to the neural control of swallowing (Kumar et al., 2012; Ko et al., 2019; Jang et al., 2020a,b), also paralleling related literature on bilateral gray matter damage and dysphagia (Ickenstein et al., 2003).

Finally, two studies (Galovic et al., 2013, 2017) indicated that disruptions in white matter connections are particularly disruptive to swallowing in the early post-stroke phase (i.e., within a week post stroke), but have quick recovery potential and can help restore swallowing function. The critical role of white matter in swallowing recovery was further highlighted in two additional studies that showed swallowing recovery upon white matter tracts adaptations post stroke (Mihai et al., 2016; Jang et al., 2017). These studies prompt questions surrounding the critical role that white matter plasticity may play in swallowing recovery. They also identify an area in need of rigorous exploration and with high potential impact for swallowing recovery and rehabilitation.

### Limitations

A systematic review is always limited by the available evidence. We identified only twenty-two studies that met our inclusion criteria, even though our inclusion criteria were rather broad. Another limitation is that meta-analysis was not possible due to heterogeneous study designs, and all synthesis of findings was qualitative. Finally, in order to synthesize data, we used a quality assessment, but we had to modify the most relevant quality assessment available because not all components applied to this type of observational research, which we acknowledge introduces some bias.

### Conclusion

This systematic review highlighted the critical role of white matter in the neural control of swallowing, which is an area that has been significantly understudied. Findings indicated that white matter damage can be directly tied to swallowing deficits, and several white matter tracts (such as the pyramidal tracts, internal capsule, superior longitudinal fasciculus, corona radiata, corpus callosum, and external capsule) were implicated across studies. Despite these findings, several methodological limitations were also identified in most reviewed studies and need to be addressed in the future. It is our hope that this systematic review will serve as a starting point for future research that will build a more thorough understanding of the role of white matter in the neural control of normal swallowing, and, more critically, will inspire future work on delineating its role in dysphagia recovery and rehabilitation.

## Data Availability Statement

The raw data supporting the conclusions of this article will be made available by the authors, without undue reservation.

## Author Contributions

GM conceptualized the study, study design, and is the guarantor of the study. GM, AA, and RH designed the study and wrote the article. BM designed and performed the search, with input from all other authors. HC provided input on the methods. AA and RA screened, read, and assessed all studies. GM resolved disagreements and trained AA and RA in qualitative review. All authors read and revised manuscript drafts, and approved the final manuscript version.

## Conflict of Interest

The authors declare that the research was conducted in the absence of any commercial or financial relationships that could be construed as a potential conflict of interest.

## Abbreviations

CNS: central nervous system
NGT: nasogastric tube
DWI: diffusion weighted imaging
FA: fractional anisotropy
TV: tract volume
VFSS: videofluoroscopic swallow study
PAS: penetration aspiration scale
FOIS: functional oral impact scale
CBT: corticobulbar tract
CD: cannot determine
GUSS: Gugging Swallow Screen
LA: leukoaraiosis (hyperintensity around ventricles)
ASHA: NOMS American Speech Language Hearing Association National Outcome Measurement System
OR: odds ratio
WM: white matter
MCA: middle cerebral artery
NIHSS: national institute of health stroke scale
VLSM: voxel based lesion symptom mapping
ROI: regions of interest
MBSIMP^©TM^: Modified Barium Swallow Impairment Profile
CNS: Canadian Neurological Scale
MRI: magnetic resonance imaging
SLP: speech language pathologist
NR: Not Rated
FEES: Fiberoptic Endoscopic Evaluation of Swallowing
GMFCS: gross motor function classification scale
MACS: manual ability classification scale
RD: radial diffusivity
MD: mean diffusivity
FC: fibers count
DDS: Dysphagia Disorder Survey
DMSS: dysphagia management staging scale
fMRI: functional magnetic resonance imaging
BODS-2: Bogenhausen Dysphagia Score Part 2
FEDSS: fiberoptic endoscopic dysphagia severity scale
SMA: supplementary motor area
PVWM: periventricular white matter.
