# Supplementary material for: The Role of White Matter in the Neural Control of Swallowing: A Systematic Review
Source: Front Hum Neurosci. 2021 Jun 28;15:628424. doi: 10.3389/fnhum.2021.628424 (PMC8273764; doi:10.3389/fnhum.2021.628424)
Supplement: Supplementary file 1 [file Table_1.docx]

**Supplemental Table A: Full Search Strategy**

| **Data Base** | **Search Strategy** | **Time of search** | **Results** |
| --- | --- | --- | --- |
| Medline via PubMed | (("Magnetic Resonance Imaging"[Mesh] OR MRI OR Magnetic Resonance Imaging) AND ("White Matter"[Mesh] OR white matter) AND ("Deglutition Disorders"[Mesh] OR Deglutition Disorders OR Dysphagia)) | July 10, 2020  November 8, 2020 | 88  89 |
| CINAHL | Search Strategy: ((MRI OR Magnetic Resonance Imaging) AND (white matter) AND (Deglutition Disorders OR Dysphagia)) | July 10, 2020  November 8, 2020 | 27  24 |
| *Web of Science Core Collection | ((MRI OR Magnetic Resonance Imaging) AND (white matter) AND (Deglutition Disorders OR Dysphagia)) | July 10, 2020  November 8, 2020 | 71  73 |
|  | Back-chained search of all reference lists of selected articles. | July 29, 2020  November 8, 2020 | 2  0 |

*Web of Science Core Collection (includes the following: Science Citation Index Expanded (1900-present); Social Sciences Citation Index (1900-present); Arts & Humanities Citation Index (1975-present); Conference Proceedings Citation Index- Science (1990-present); Conference Proceedings Citation Index- Social Science & Humanities (1990-present); Book Citation Index– Science (2005-present); Book Citation Index– Social Sciences & Humanities (2005-present); Current Chemical Reactions (1985-present); Index Chemicus (1993-present); and Emerging Sources Citation Index (2005 – present)

On Nov 8 there was 3 novel articles none of which met the criteria

Database Searched: Cochrane Library Database of Systematic Reviews

Note: No similar systematic reviews were identified for this topic in the Cochrane Library Database of Systematic Reviews.
