# Supplementary material for: The Role of White Matter in the Neural Control of Swallowing: A Systematic Review
Source: Front Hum Neurosci. 2021 Jun 28;15:628424. doi: 10.3389/fnhum.2021.628424 (PMC8273764; doi:10.3389/fnhum.2021.628424)
Supplement: Supplementary file 2 [file Table_2.docx]

**Supplemental Table B:** Modified NIH Quality Assessment Form for observational cohort and cross-sectional studies.  *Original tool available at:* [*https://www.nhlbi.nih.gov/health-topics/study-quality-assessment-tools*](https://www.nhlbi.nih.gov/health-topics/study-quality-assessment-tools)

| **Assessing the Quality of Observational Cohort and Cross-Sectional Studies**  *Rate: Yes, No, P (partially) CD (cannot determine), NA (not applicable), or NR (not reported). Then add details for justification in the blank box below.* | |
| --- | --- |
| **1. Research question**  Was the research question or objective in this paper clearly stated? | Yes/No/P/CD/NA/NR |
| Did the authors describe their goal in conducting this research?  Is it easy to understand what they were looking to find? |  |
| **2 & 3. Study population**  Was the study population clearly specified? | Yes/No/P/CD/NA/NR |
| Did the authors describe the group of people from which the study participants were selected or recruited, using demographics, location, and time period?  If you were to conduct this study again, would you know who to recruit, from where, and from what time period?  Is the cohort population free of the outcomes of interest at the time they were recruited?  **# 3 Was the population rate at least 50%**  Please note this information  *If fewer than 50% of eligible persons participated in the study, then there is concern that the study population does not adequately represent the target population. This increases the risk of bias.* |  |
| **4. Groups recruited from the same population and uniform eligibility criteria**  Were all the subjects selected or recruited from the same or similar populations (including the same time period)? Were inclusion and exclusion criteria for being in the study prespecified and applied uniformly to all participants? | Yes/No/P/CD/NA/NR |
| Were the inclusion and exclusion criteria developed prior to recruitment or selection of the study population?  Were the same underlying criteria used for all of the subjects involved? |  |
| **5. Sample size justification**  Was a sample size justification, power description, or variance and effect estimates provided? | Yes/No/P/CD/NA/NR |
| Did the authors present their reasons for selecting or recruiting the number of people included or analyzed?  Do they note or discuss the statistical power of the study? *This question is about whether or not the study had enough participants to detect an association if one truly existed.* |  |
| **6. Primary diagnosis determined prior to outcome measurement.** | Yes/No/P/CD/NA/NR |
| For the analyses in this paper, were the diagnosis/disorders/exposure(s) of interest measured prior to the outcome(s) being measured? |  |
| **7. Sufficient timeframe to see an effect** Did the study allow enough time for a sufficient number of outcomes to occur or be observed, or enough time for an exposure to have a biological effect on an outcome? | **N/A** |
| **8. Different levels/severity of the diagnosis.**  For diagnoses/disorders/exposures that can vary in amount or level, did the study examine different levels as related to the outcome (e.g., categories of diagnosis/disorders/exposure, or measured as continuous variables)? | Yes/No/P/CD/NA/NR |
| If the diagnosis/disorders/exposure can be defined as a range (examples: drug dosage, amount of physical activity, amount of sodium consumed), were multiple categories of that exposure assessed? |  |
| **9. Diagnosis measures and assessment**  Were the diagnoses/disorders/exposure measures (independent variables) clearly defined, valid, reliable, and implemented consistently across all study participants? | Yes/No/P/CD/NA/NR |
| Were the diagnosis/disorders/ exposure measures defined in detail?  Were the tools or methods used to measure exposure accurate and reliable–for example, have they been validated or are they objective? |  |
| **10. Repeated exposure assessment** | **N/A** |
| **11. Outcome measures**  Were the outcome measures (dependent variables) clearly defined, valid, reliable, and implemented consistently across all study participants? | Yes/No/P/CD/NA/NR |
| Were the outcomes defined in detail?  Were the tools or methods for measuring outcomes accurate and reliable–for example, have they been validated or are they objective? |  |
| **12. Blinding of outcome assessors**  Were the outcome assessors blinded to the diagnoses/disorders/exposure status (i.e. dysphagia) of participants? | Yes/No/P/CD/NA/NR |
| *Sometimes the person measuring the exposure is the same person conducting the outcome assessment. In this case, the outcome assessor would most likely not be blinded to exposure status because they also took measurements of exposures. If so, make a note of that in the comments section.*  If blinding was impossible for clinical reasons, mark "NA" and explain potential bias. |  |
| **13. Follow-up rate** | **N/A** |
| **14. Statistical analyses**  Were key potential confounding variables measured and adjusted statistically for their impact on the relationship between exposure(s) and outcome(s)? | Yes/No/P/CD/NA/NR |
| Were key potential confounding variables measured and adjusted for, such as by statistical adjustment for baseline differences (i.e. sex, age, other diagnoses)? |  |
