# Supplementary material for: The Role of White Matter in the Neural Control of Swallowing: A Systematic Review
Source: Front Hum Neurosci. 2021 Jun 28;15:628424. doi: 10.3389/fnhum.2021.628424 (PMC8273764; doi:10.3389/fnhum.2021.628424)
Supplement: Supplementary file 3 [file Table_3.DOCX]

**Supplemental Table C:** Extended summary of participant information, methods, and major findings and quality of studies included in qualitative synthesis (n=20 studies)

|  | Participant Information | | |  | Methods | | |  | | Findings and Quality | | | |
| --- | --- | --- | --- | --- | --- | --- | --- | --- | --- | --- | --- | --- | --- |
| Study Type | Number of participants, age & sex | Underlying diagnosis | |  | White Matter Method* | Swallowing Measurement *method* | Swallowing *analysis* | |  | Implicated WM areas | Associations with swallowing | Limitations | NIH Quality |
| Jang 2020 b  Retrospective Cohort | n = 40  20 controls: 56 ± 10 (55% female)  16 pts w/ dysphagia and NGT < 6 mos: 60.6 ± 12.4 (37.5% female)  4 pts w/ dysphagia and NGT > 6 mos: 63.2 ± 14.7 (75% female) | | Stroke: Lateral medullary infarct with dysphagia and NGT placement |  | DWI  FA  TV  Fazekas grade | VFSS | PAS; FOIS | |  | Corticobulbar tract | FA in CBT was significantly reduced in pts w/ longer compared to shorter NGT use or controls (p < 0.05), but was not significantly different between patients w/ dysphagia and NG for <6 mos and controls  Fazekas ratings were not significantly different between patient groups | CD blinding; did not account for confounding variables in statistical analyses; no sample size justification | Fair |
| Jang 2020 a  Retrospective Cohort | n = 64  22 controls: 51 ± 11 (50% female)  10 pts w/ dysphagia and NGT < 2 days, 51 ± 15 (40% female)  27 pts w/ dysphagia and NGT < 6 mos. 60 ± 8.9 (52% female)  5 pts w/ dysphagia and NGT > 6 mos. 59 ± 10 (40% female) | | Stroke: Supratentorial intra-cerebral and intra-ventricular hemorrhage with dysphagia and NGT placement |  | DWI  FA  TV | GUSS, VFSS | PAS; residue scale | |  | Corticobulbar tract | Patients who had NGT removed within 2 days had milder CBT injury (only FA decreased, not TV)  FA in CBT for each patient group was lower than controls (p < 0.05)  CBT TV was lower than controls in both hemispheres for patients with NGT <6 mos, and lower for both hemispheres for patients with NGT > 6 mos  TV of the CBT in the affected hemisphere was negatively correlated with length of time until NGT removal in < 6 mos group (r = 0.430, p < 0.05)  They were able to remove NGT within 6 mos for individuals with unilateral injuries but, not for individuals with bilateral injuries | CD blinding; did not account for confounding variables in statistical analyses; no sample size justification | Fair |
| Lee 2020  Retrospective Cohort | n = 137  68.7 ± 14  49.6% female | | Stroke: acute ischemic stroke and referred for VFSS |  | DWI; FLAIR  Fazekas grade | VFSS | Clinical dysphagia scale | |  | White matter; corona radiata/internal capsule/basal ganglia | Bilateral lesions at the corona radiata/internal capsule/basal ganglia were significant prognostic factors for persistent dysphagia (p < 0.001). | CD blinding; no sample size justification; no details on white matter or swallowing analysis methods; collapsing brain areas into one category | Fair |
| Ko 2019  Case control | n = 81  64.6±11.5  36% female | | Stroke: First unilateral corona radiata infarct involving CBT |  | NR  Lesion size, Fazekas grade | Clinical swallow, VFSS for 5/20 case and 11/61 comparison | Feeding method at discharge, "clinical judgement" of swallow, videofluoroscopic dysphagia scale, PAS, ASHA NOMS, oral transit time, pharyngeal transit time | |  | Corticobulbar tract | LA involving contralateral CBT was a significant predictor of feeding method at discharge (b = -3.95, OR=0.02, p < 0.01) and NOMS score (b = 1.56, p = 0.03). | Limited information on WM assessment, Age was a confounding factor, no sample size justification, inconsistent use of video swallowing | Fair |
| Wilmskoetter 2019  Retrospective Cohort | n = 68  68.21± 15.23  53% female | | Stroke: First (and only) hemispheric stroke of the MCA |  | DWI  VLSM  ROI | VFSS | Modified Barium Swallow Impairment Profile and PAS | |  | Corona radiata, superior longitudinal fasciculus, external capsule, ansa lenticularis, lenticular fasciculus. | Regions surviving corrected threshold (z <-2.78) for impaired laryngeal elevation: Right external capsule (8.2%), right superior longitudinal fasciculus (0.1%), right superior corona radiata (0.2%). Regions surviving corrected threshold (z <-2.78) for ROI for anterior hyoid excursion: left ansa lenticularis, left lenticular fasciculus. Regions surviving corrected threshold (z <-3.43) for laryngeal vestibule closure: right superior corona radiata (0.1%), right external capsule (5.5%). Regions surviving corrected threshold (z <-3.33) for pharyngeal residue: right superior corona radiata (1.2%), right posterior corona radiata (12.5%), right tapatum (1.7%), posterior limb of right internal capsule (0.2%), retrolenticular part of right internal capsule (3.9%), right superior longitudinal fasciculus (8.8%). Regions surviving corrected threshold (z <-4.36) for PAS: right superior longitudinal fasciculus (1.7%). | Statistical power higher in some brain regions (more damaged areas), missing data, limited discussion of power, CD blinding of outcome assessors. | Good |
| Fandler 2018  Retrospective Cohort | n = 243  67.7± 11.9 (45.5% female)  47 pts w/ dysphagia 70.8± 10.8 (30% female)  196 pts wo/ dysphagia 67.1± 12.4 (37.2% female) | | Stroke: Recent small subcortical infarct |  | Lesion probability | GUSS | SLP GUSS rating: absent, mild, moderate, severe | |  | Pyramidal tract (unspecified); contralateral lacune (unspecified); contralateral WM hypersensitivities (unspecified). | All patients with moderate or severe dysphagia had damage along the pyramidal tract, compared to 86% of patients without dysphagia. Patients with moderate to severe dysphagia more frequently had damage to the pyramidal tract and a contralateral pyramidal tract lacune (77.8% vs. 19.9%, p < 0.001) | Limited details on WM analysis, used a screener to identify dysphagia, did not incorporate confounds in stats, no sample size justification, | Fair |
| Fandler 2017  Retrospective Cohort | n = 322  249 pts wo/ dysphagia: 66.8 ± 12.5 (37.4% female)  83 pts w/ dysphagia 70.2 ±9.9 (30% female) | | Stroke: Recent small subcortical infarct |  | MRI, DWI  Manual Lesion identification and Fazekas grade | GUSS | SLP GUSS rating: absent, mild, moderate, severe | |  | Unspecified | More severe WM hyperintensities were an independent predictor of dysphagia *(p* < 0.03), but that association was lost when analysis was restricted to patients with supratentorial damage (*p* < 0.27). | Screener for dysphagia, unspecified WM locations, no sample size justification, CD blinding for swallowing. | Fair |
| Flowers 2017  Retrospective Cohort | n = 160  66.7 ± 15 (43.1% female)  84 pts wo/ dysphagia: 63.6 ± 15.6 (46.4% female)  76 pts w/ dysphagia: 69.9±13.8, (39.5% female) | | Stroke |  | MRI, DWI  ROI  Fazekas grade | Clinical or instrumental evaluation by SLPs, or radiographic confirmation of enteral feeding. | NR | |  | Internal capsule, | Internal capsule had an OR = 2.9 for dysphagia. | Did not give assessment details for swallowing, no sample size justification, heterogeneous sample, broadly defined regions, CD blinding. | Fair |
| Galovic 2017  Cohort | n = 86:  62 Patients: 75 ± 21 years (55% female).  24 Controls: 63± 9 (42% female) | | Stroke: First hemispheric stroke leading to impaired oral intake |  | MRI, DWI  VLSM  ROI  Probabilistic Tractography | Clinical Swallow. If results of clinical swallow were "indeterminate" performed FEES. Swallowing eval completed baseline and in >- 7 days. 4 Week follow-up was a structed interview on diet. | Clinical Swallow (may have included an assortment of: 50 mL swallow test, Any 2 Scale, Gugging, Functional Oral Intake Scale). They used specific criteria from the assorted scales to rate FOIS level 1-7. | |  | Superior Corona Radiata, superior longitudinal fascicle, external capsule. Probabilistic tractography: projection fibers (thalamic and cortico-bulbar), lesser degree commissural fibers (to the contralateral thalamus) | Statistical map of voxels associated with impaired oral intake after 7 days affected 89% WM with a center of maximum overlap over superior corona radiata with location immediately anterior to facial fibers (65% superior corona radiata, 12% superior longitudinal fascicle, 8% external capsule). Proportion of damaged voxels in the superior corona radiata was negatively correlated with degree of oral intake after 7 days (*p* = 0.001). After four weeks, the statistical lesion map covered 76% grey matter. | No classification of stroke severity, telephone assessment for last phase, no sample size justification. | Fair |
| Jang 2017  Single Case Study | n = 1  59  Male | | Stroke |  | DWI  ROI  Probabilistic Tractography | NR | Severe dysphagia, fed by Levin tube | |  | Corticobulbar tract | At the 5-week follow-up, the right CBT was discontinued at the subcortical right matter (Severe narrowing), left was not reconstructed. Right CBT recovered after rehabilitation and cranioplasty: thickened and extended to cerebral cortex. Resolution of dysphagia. | No details on swallowing measurement, limited demographic details, no discussion of power, no statistical analysis, CD blinding. | Fair |
| Moon 2017  Retrospective Cohort | n = 63  77.24 ± 7.22  50.8% female | | Stroke: mild first stroke, had dysphagia symptoms and completed VFSS. |  | MRI, DWI  Fazekas grade | Logemann VFSS procedure | Clinician description of swallowing elements and measurement of oral transit time, swallowing response time, pharyngeal transit time in seconds, and penetration/aspiration | |  | Unspecified | WM lesions are correlated with prolonged oral transit time (r = 0.384, p = 0.003) and increased penetration (r = 0.322, p = 0.015), even controlling for confounding variables. Mean oral transit time (OR = 3.082, p = 0.03) and penetration (OR = 2.521 p = 0.015) were significantly different in the severe WM lesion group than in the mild group. Left lesions associated with mastication (p = 0.039). | Unspecified WM lesions, subjective measures, no specific hypotheses, limited validated swallowing outcomes, no reliability testing, no sample size justification, CD blinding for swallow assessment. | Fair |
| Mourão 2017  Cohort | n = 20  5.11-17.6  45% female | | Unilateral spastic cerebral palsy |  | MRI, DWI  FA RD MD FC | Clinical Swallow | DDS, DMSS | |  | Anterior, middle, posterior corpus callosum | Left hemisphere group (less severe): As FA (r = -0.667, p = 0.013) and FC decreased (r =-0.829, p < 0.001) and RD increased (r = 0.594, p = 0.032) (i.e. reduced structural integrity of the corpus callosum), dysphagia increased. Reduced FC in middle (r = -0.762, p = 0.002) and posterior (r= -0.739, p = 0.004) CC was associated with increased (worse) DDS. Right hemisphere group was more severe, and no significant correlations were observed. | Heterogeneous groups did not account for confounding variables in statistical analyses, no sample size justification. | Fair |
| Galovic 2016  Cohort | n = 119  107 Tube independent: 71± 19 (45% female)  12 Tube dependent: 76+- 9 (50% female) | | Stroke: first hemispheric stroke who failed dysphagia screening |  | MRI  VLSM | Clinical Swallow | BODS-2 | |  | Superior corona radiata, external capsule, superior longitudinal fascicle | Mildly impaired oral intake was correlated with a widespread grey and white-matter network shown by a statistical map that included the superior corona radiata (12%), the external capsule (10%) and the superior longitudinal fascicle (8%). | No discussion of blinding, no sample size justification, limited accounting for confounding variables | Good |
| Mihai 2016  Case Control | n = 36  18 Patient: 56.6± 15.3 (27.8% female)  18 Control: 61.94± 9.78 (77.8% female) | | Stroke: Single ischemic stroke (recovered from Severe dysphagia w/in last 3 years) |  | fMRI DWI  FA  Lesion size | Clinical Swallow and VFSS for a limited subset | BODS-2, Neurogenic Oral Dysphagia test, water swallowing test (dysphonia, dysarthria, impaired cough, gag, coughing/change in voice) | |  | Pyramidal track laterality (between tongue and posterior limb of internal capsule). | Overall laterality of fractional anisotropy differed between patients and controls (t(32) = 3.21, p <0.005). Patients showed asymmetric laterality of the pyramidal tract between tongue area and posterior limb of internal capsule. The larger the lesion, the more asymmetric (r = -0.676, p < 0.001). Laterality index was positively associated with compliance, meaning less compliant patients were less symmetric (r = 0.65, p < 0.009). | Lesions were heterogeneous, not all patients had VFSS, they each had individualized therapy which varied, hard to control for compliance, CD blinding. | Good |
| Wan 2016  Retrospective Cohort | n = 12  66 ±10  25% female | | Stroke: basal ganglia and/or centrum semiovale |  | MRI  NR | VFSS and FEES | VFSS: residue, oral transit time, tongue base and pharyngeal wall contact, pharyngeal delay, delayed laryngeal elevation, timing and %, FEES: asymmetry/degree of muscle contraction, vocal fold movement, residue, cricopharyngeal atrophy | |  | Centrum semiovale in conjunction with basal ganglia | 83% of 12 patients had dysphagia. | No hypotheses, Small heterogeneous sample, no sample size justification, no diagnosis levels of severity, no MRI information, minimal participant information, no blinding, no confounding variables in analysis. | Poor |
| Suntrup 2015  Cohort | n = 200  73.7 years  49.5% female  W/o dysphagia (72±10.7)  W/ mild dysphagia (73.5± 13.1)  W/ severe dysphagia (74.8± 11.9) | | Stroke: First, failed dysphagia screening and completed FEES |  | MRI, DWI  Atlas-based regional lesion analysis | FEES | FEDSS ranking1-6. | |  | Superior longitudinal fasciculus (right), superior longitudinal fasciculus temporal part (right), corticospinal tract (right). | Patients with dysphagia had a significant difference of mean percentage lesioned volume in the following areas: superior longitudinal fasciculus (right; p < 0.021, OR = 4.52), superior longitudinal fasciculus temporal part (right; p < 0.028, OR = 4.17), corticospinal tract (right; p < 0.044, OR = 2.79). | No sample size justification, limited methodology for WM and swallowing measures, unclear MRI settings, no confounding variables in analysis. | Fair |
| Kim 2014  Retrospective Cohort | n = 103  NR  NR | | Stroke: First unilateral ischemic stroke within 3 months, dysphagia symptoms completed VFSS in last three months |  | MRI  Vascular white matter territory | VFSS (Logemann procedure) | Lip closure, oral residue, bolus formation, oral transit time, delay of trigger, height of laryngeal elevations nasal regurgitation, residue in Valleculae/pyriform sinus, pharyngeal delay time, pharyngeal transit time, penetration, aspiration | |  | Unspecified | Excessive vallecular residue observed most frequently in the WM group (*p* < 0.002) | Unspecified WM lesions, no participant information, no assessment of stroke severity, not able to replicate, no MRI specifications, no sample size justification, no confounding variables in analyses. CD blinding. | Poor |
| Li 2014  Cohort | n = 36  12 w/o dysphagia 66.5 ± 5.2 (41.7% female)  12 w/ dysphagia: 65.2 ± 4.3 (50% female)  12 Control 65.8±3.3 (50% female) | | Stroke: First (and only) hemispheric stroke of the MCA |  | Resting state fMR  DWI  ROI, FA, seed-based connectivity maps | Clinical Swallow and VFSS. | SLP tracked Logemann's indicators: cough, oral residue, delayed swallow, reduced laryngeal elevation, throat clearing, choking. VFSS: PAS. Dysphagia = one of: 50% of vallecular/ pyriform residue, aspiration, pharyngeal transit time >2 seconds, impaired cricopharyngeal relaxation. | |  | Bilateral Corticospinal tract, Corpus Callosum | Reduced FA for left SMA to right SMA (corpus callosum), left SMA to internal capsule (corticospinal tract), and right SMA to internal capsule (corticospinal) in pts with dysphagia compared to controls. There were clear differences between stroke patients with dysphagia and healthy controls, but not between patients with and without dysphagia. | Limited detail on MRI, No sample size justification, no stroke severity classification, CD blinding, no confounding variables in statistical analyses. Only MCA dysphagia, placed seeds for WM tracking closer to the cortex. | Fair |
| Galovic 2013  Prospective Cohort | n = 94  34 acute risk: 74 ± 19 (59% female),  60 no risk: 71.5 ±16 (43% female) | | Stroke: first |  | MRI, DWI  ROI lesion mapping | Clinical Swallow | Daniels et al. standardized scale for aspiration risk (6 features) and BODS-2 functional oral intake scale. | |  | Internal capsule, PVWM | Acute findings: internal capsule (OR = 7.6, p < 0.001), periventricular WM (OR = 4.8, p < 0.001). When adjusted for NIHSS and lesion size, internal capsule (OR = 6.2, p < 0.002) and now PVWM OR = 2.7, p < 0.06). Model accuracy 76%. No WM areas were associated with extended risk of aspiration assessed at 7 days (only the cortical area of frontal operculum). | No sample size justification, CD blinding, Clinical "risk of aspiration" with no instrumental, no mild strokes, only analyzed early subacute phase. | Good |
| Kumar 2012  Retrospective cohort | n = 77  Median = 76  (64.9% female) | | Stroke: Acute Ischemic Stroke and severe dysphagia |  | DWI  Lesion volume | Clinical and/or video swallow | Classified severe dysphagia as complete absence of oral intake recommended by SLP or presence of significant aspiration on tasks. | |  | Unspecified | In univariate analysis PVWM was not a significant predictor of PEG placement in severely dysphagic stroke patients. However, in a multivariate analysis with age, NIHSS score, lesion volume, and brain locations, PVWM approached significance (p < 0.057 with OR = 3.829). NIHSS score and Bihemispheric lesions were significant predictors, but PVWM was more than any other individual area (insula or IFG) | Unspecified white matter lesions, only participants with severe dysphagia, limited details on measurement methods, no sample size justification, CD blinding. | Fair |
| Cola 2010  Case Control | n = 45  10 RHD 62.3 ± 12.2 (10% female)  10 LHD 62.3±8.7 (0% female)  25 Control 67.2± 9.1(8% female) | | Stroke: Acute unilateral ischemic subcortical stroke |  | DWI  Lesion volume | VFSS | Bolus timing (oral transit time, stage transit duration, pharyngeal response time), PAS, bolus clearance (vallecular retention and pyriform sinus retention). Classified as dysphagia if at least 2 scores >2 standard deviations from mean | |  | Unspecified | Significant interaction between peri-ventricular WM lesions and hemisphere (chi square = 9.85, p = 0.002). 100% had dysphagia in LH PVWM group, 0% dysphagia for RHD. No association was detected for those without PVWM damage. | Unspecified WM lesions, Small n, no MRI in healthy subjects, controls were not matched for race, no sample size justification, | Good |
| Levine 1992  Cohort | 49 controls  Mean = 66  (40.8% female) | | n/a |  | MRI  MRI Score | VFSS | Oral transit time, stage transition duration, pharyngeal response duration, and total swallow duration. | |  | Unspecified | Total swallow duration (p < 0.009) and oral transit duration (p < 0.047) significantly differed by MRI score (total number of WM unidentified bright objects). | Unspecified WM lesions only healthy controls, no sample size justification, limited details on participants, old imaging methods. | Fair |

**Abbreviations in order of appearance:** NGT nasogastric tube, DWI diffusion weighted imaging, FA fractional anisotropy, TV tract volume, VFSS videofluoroscopic swallow study, PAS penetration aspiration scale, FOIS functional oral impact scale, CBT corticobulbar tract, CD cannot determine, GUSS Gugging Swallow Screen, LA leukoaraiosis (hyperintensity around ventricles), ASHA NOMS American Speech Language Hearing Association National Outcome Measurement System, OR odds ratio, WM white matter, MCA middle cerebral artery, NIHSS national institute of health stroke scale, VLSM voxel based lesion symptom mapping, ROI regions of interest, MBSImP Modified Barium Swallow Impairment Profile, CNS Canadian Neurological Scale, MRI magnetic resonance imaging, SLP speech language pathologist, NR Not Rated, FEES Fiberoptic Endoscopic Evaluation of Swallowing, GMFCS gross motor function classification scale, MACS manual ability classification scale, RD radial diffusivity, MD mean diffusivity, FC fibers count, DDS Dysphagia Disorder Survey, DMSS dysphagia management staging scale, fMRI functional magnetic resonance imaging, BODS-2 Bogenhausen Dysphagia Score Part 2, FEDSS fiberoptic endoscopic dysphagia severity scale, SMA supplementary motor area, PVWM periventricular white matter.

*See Table 3 for detailed white matter methodology
